# Eigenvalue Estimates on Weighted Manifolds

**DOI:** 10.1007/s00025-024-02214-3

**Published:** 2024-06-16

**Authors:** Volker Branding, Georges Habib

**Affiliations:** 1 Faculty of Mathematics, University of Vienna, Oskar-Morgenstern-Platz 1, 1090 Vienna, Austria; 2 Department of Mathematics, Faculty of Sciences II, Lebanese University, P.O. Box 90656, Fanar-Matn, Lebanon; 3 CNRS, IECL, Université de Lorraine, F-54506 Nancy, France

**Keywords:** Hodge Laplacian, weighted manifold, Jacobi operator, eigenvalue estimates, 35P15, 53C42, 58J50

## Abstract

We derive various eigenvalue estimates for the Hodge Laplacian acting on differential forms on weighted Riemannian manifolds. Our estimates unify and extend various results from the literature and provide a number of geometric applications. In particular, we derive an inequality which relates the eigenvalues of the Jacobi operator for *f*-minimal hypersurfaces and the spectrum of the Hodge Laplacian.

## Introduction and Results

The aim of spectral geometry is to obtain deep insights into both topology and geometry of Riemannian manifolds from the spectrum of differential operators. However, in the case of an arbitrary Riemannian manifold, it is in general not possible to explicitly calculate the spectrum of a given differential operator. For this reason, it is very important to obtain characterizations of the eigenvalues of such operators in terms of geometric data as for example curvature.

The most prominent spectral problem one can study is the case of the Laplace operator acting on functions for which we recall several important results. Let $\Omega \subset M$ be a bounded domain of a Riemannian manifold *M* with smooth boundary $\partial \Omega$ and let $\lambda$ be an eigenvalue of the Dirichlet problem

1.1 $$\begin{aligned} \left\{ \begin{array}{ll} \Delta u=\lambda u\,\, &{}\text {on}\,\, \Omega \\ u=0 \,\,&{}\text {on}\,\, \partial \Omega \end{array}\right. \end{aligned}$$

where $u\in C^\infty (M)$ and $\Delta$ is the Laplace-Beltrami operator. From general spectral theory, we know that the spectrum of the Dirichlet problem (1.1) is discrete and that the eigenvalues satisfy

$$\begin{aligned} 0<\lambda _1\le \lambda _2\le \lambda _3\le \ldots \le \lambda _j\le \ldots \rightarrow \infty . \end{aligned}$$

In the case that the manifold *M* is the Euclidean space with the flat metric $(\mathbb {R}^n,\textrm{can})$, Payne, Pólya and Weinberger [29, 30] derived the following so-called *universal inequalities* for the Dirichlet problem of the Laplace operator (1.1). Namely, they show that for any integer $k\ge 1$

1.2 $$\begin{aligned} \lambda _{k+1}-\lambda _k\le \frac{4}{nk}\sum _{i=1}^k\lambda _i. \end{aligned}$$

An immediate consequence of (1.2) is the following inequality,

$$\begin{aligned} \lambda _{k+1}\le \left( 1+\frac{4}{n}\right) \lambda _k, \end{aligned}$$

which allows to get an estimate of all the eigenvalues if an upper bound is known on the lowest one $\lambda _1$. Another kind of such inequality has been further developed by Hile and Protter (see [19]) and by Yang in [40] who established the following estimate

1.3 $$\begin{aligned} \sum _{i=1}^k(\lambda _{k+1}-\lambda _i)^2\le \frac{4}{n}\sum _{i=1}^k(\lambda _{k+1}-\lambda _i)\lambda _i. \end{aligned}$$

This inequality improves all the previous estimates. In [11], Cheng and Yang were able to turn the recursion formula (1.3) into an estimate for the $(k+1)$-th eigenvalue of (1.1) in terms of some power of *k* as follows

1.4 $$\begin{aligned} \lambda _{k+1}\le C_0(n,k)k^\frac{2}{n}\lambda _1, \end{aligned}$$

where $C_0(n,k)$ is a constant only depending on *n* and *k*. For more details on the spectral properties of the Dirichlet problem (1.1) we refer to the lecture notes [2].

Many of these formulas have been generalized to the case of a Riemannian manifold. In this setup, Chen and Cheng [9] obtained an extrinsic estimate for eigenvalues of the Dirichlet problem (1.1) of the Laplacian on a complete Riemannian manifold $(M^n,g)$ that is isometrically immersed in an $(n+m)$-dimensional Euclidean space $\mathbb {R}^{n+m}$ (see also [14]). The estimate is

1.5 $$\begin{aligned} \sum _{i=1}^k(\lambda _{k+1}-\lambda _i)^2\le \frac{4}{n}\sum _{i=1}^k(\lambda _{k+1}-\lambda _i)\left( \lambda _i+\frac{n^2}{4}\sup _\Omega |H|^2\right) . \end{aligned}$$

Here, $H:=\frac{1}{n}\textrm{trace}(\textbf{II})$ denotes the mean curvature of the immersion and $\textbf{II}$ is the second fundamental form. As a consequence, they deduce an upper bound for the $(k+1)$-th eigenvalue in terms of a power of *k* that involves the mean curvature.

In this article, we focus on the study of geometric differential operators on an interesting class of Riemannian manifolds, the so-called *Bakry-Emery manifolds*. A Bakry-Emery manifold is a triple $(M,g,d\mu _f)$, where instead of the Riemannian measure $dv_g$ one considers the weighted measure $d\mu _f:=e^{-f}dv_g$ with $f\in C^\infty (M)$. One often refers to this kind of manifolds as *weighted manifolds*. While the initial motivation to study such kind of manifolds was to model diffusion processes [4] they have by now become famous in the study of self-similar solutions of the Ricci flow, the so-called *Ricci solitons*. Also they appear in the analysis of *shrinkers*, which represent a special class of solutions of the mean curvature flow. We refer to [13, Section 2] and [21] for more details.

Due to the presence of the weight, differential operators on weighted manifolds contain additional contributions and also their spectrum is different compared to the case of standard Riemannian manifolds. For example, the Laplace operator acting on functions on a weighted manifold is defined as follows

1.6 $$\begin{aligned} \Delta _f:=\Delta +\nabla _{df}, \end{aligned}$$

where $\Delta =\delta d$ is the standard Laplace-Beltrami operator. A direct computation shows that $\Delta _f$, usually called *drift Laplacian*, is elliptic and self-adjoint (if *M* is compact) with respect to the weighted measure $e^{-f}dv_g$. Therefore, its spectrum is discrete and consists of an increasing sequence of eigenvalues

$$\begin{aligned} 0<\lambda _{1,f}\le \lambda _{2,f}\le \dots \le \lambda _{j,f}\le \dots \rightarrow \infty . \end{aligned}$$

As in the standard case, the eigenvalue 0 corresponds to constant functions. Several results have been obtained on the spectrum of this operator (see for example [26–28]) of which we give a non-exhaustive list below. In particular, when a compact Bakry-Emery manifold $(M^n,g)$ is isometrically immersed into the Euclidean space, Xia and Xu [39] established the following recursion formula

$$\begin{aligned} \sum _{i=1}^k(\lambda _{k+1,f}-\lambda _{i,f})^2&\le \frac{1}{n}\sum _{i=1}^k(\lambda _{k+1,f}-\lambda _{i,f}) \bigg (4\lambda _{i,f} +4\sup _\Omega |\nabla f|\sqrt{\lambda _{i,f}}\\&\quad +n^2\sup _{\Omega }|H|^2 +\sup _\Omega |\nabla f|^2)\bigg ) \end{aligned}$$

which clearly reduces to Inequality (1.5) when *f* is zero. Also several explicit calculations for the drift Laplacian on self shrinkers were carried out in [6, 10, 41].

This article is devoted to the study of the Hodge Laplacian acting on differential forms on weighted manifolds, which we will often refer to as *drifting Hodge Laplacian*, and also denote it by $\Delta _f$ (see Subsection 2.2 for the definition). As for functions, the drifting Hodge Laplacian on differential *p*-forms has a discrete spectrum that entirely consists of nonnegative eigenvalues denoted by $(\lambda _{i,p,f})_i$. In particular, we will derive various eigenvalue estimates as well as different recursion formulas which will characterize the corresponding spectrum.

In the following, we present the main results of this article. First, we extend the results of [1, 35] to the case of weighted manifolds:

### Theorem 1.1

Let $(M^n,g, d\mu _f=e^{-f}dv_g)$ be a compact Bakry-Emery manifold that is isometrically immersed into the Euclidean space $\mathbb {R}^{n+m}$. For $p=1,\ldots ,n$, we let $\lambda _{1,p,f}$ be the first nonnegative eigenvalue of $\Delta _f$ on *p*-forms and $\lambda '_{1,p,f}$ the first positive eigenvalue of $\Delta _f$ on exact *p*-forms. Then, we have the following estimate

1.7 $$\begin{aligned} \lambda _{1,p,f}-\lambda _{1,p-1,f}\ge \frac{1}{p}{\mathop {\textrm{inf}}\limits _M}\left( \mathfrak {B}^{[p]}+T_f^{[p]}-\sum _{s=1}^m(\textbf{II}^{[p]}_{\nu _s})^2\right) . \end{aligned}$$

Also, we have the inequality

1.8 $$\begin{aligned} \lambda '_{1,p,f}\le \frac{1}{\textrm{Vol}_f(M)}\int _M \left( pn|H|^2-\frac{p(p-1)}{n(n-1)}\textrm{Scal}^M+\frac{p}{n}|df|^2\right) d\mu _f, \end{aligned}$$

where $\textrm{Scal}^M$ denotes the scalar curvature of *M*.

The tensor $\mathfrak {B}^{[p]}+T_f^{[p]}$ appearing in the first statement of Theorem 1.1 is the so-called *p*-Ricci tensor, see (2.6) for the precise definition. Also, $\textbf{II}^{[p]}_{\nu _s}$ is some canonical extension of the second fundamental form $\textbf{II}$ of the immersion to differential *p*-forms in the direction of the normal vector field $\nu _s$, see (3.3) for more details.

### Remark 1.2

1. It is well-known that $\lambda _{1,f}=\lambda '_{1,1,f}$ where $\lambda _{1,f}$ is the first positive eigenvalue of $\Delta _f$ on functions. Hence we get the estimate for $\lambda _{1,f}$
  1.9 $$\begin{aligned} \lambda _{1,f}\le \frac{1}{\textrm{Vol}_f(M)}\int _M \left( n|H|^2+\frac{1}{n}|df|^2\right) d\mu _f, \end{aligned}$$
  which is the same estimate as in [7].
2. It was shown in [22] that when *M* is an embedded shrinker of revolution in $\mathbb {R}^3$ such that the intersection of *M* with some sphere has only two connected components and such that *M* is symmetric with respect to reflection across the axis of revolution, then equality is attained in (1.9). Therefore, the inequality in Theorem 1.1 is attained for $p=1$.

In addition to the eigenvalue estimates presented in Theorem 1.1, we establish the following recursion formulas for the eigenvalues of the drifting Hodge Laplacian on weighted manifolds:

### Theorem 1.3

Let $X:(M^n,g)\rightarrow (\mathbb {R}^{n+m},\textrm{can})$ be an isometric immersion. For any $p\in \{0,\ldots ,n\}$, the eigenvalues of the drifting Hodge Laplacian $\Delta _f$ acting on *p*-forms on a domain $\Omega$ of *M* with Dirichlet boundary conditions satisfy for any $k\ge 1$

$$\begin{aligned} \sum _{i=1}^k(\lambda _{k+1,p,f}-\lambda _{i,p,f})^\alpha\le & {} \frac{4}{n}\sum _{i=1}^k(\lambda _{k+1,p,f}-\lambda _{i,f})^{\alpha -1}\left( \lambda _{i,p,f} -\int _\Omega \langle (\mathfrak {B}^{[p]}+T_f^{[p]})\omega _i,\omega _i\rangle d\mu _f\right. \\{} & {} \left. +\frac{n^2}{4}\int _\Omega |H|^2|\omega _i|^2d\mu _f -\frac{1}{4}\int _\Omega (2\Delta f+|df|^2)|\omega _i|^2d\mu _f\right) , \end{aligned}$$

for $\alpha \le 2$. Also, we have

$$\begin{aligned}{} & {} \sum _{i=1}^k(\lambda _{k+1,p,f}-\lambda _{i,p,f})^\alpha \\{} & {} \quad \le \frac{2\alpha }{n}\sum _{i=1}^k (\lambda _{k+1,p,f}-\lambda _{i,p,f})^{\alpha -1}\left( \lambda _{i,p,f}-\int _\Omega \langle (\mathfrak {B}^{[p]}+T_f^{[p]})\omega _i,\omega _i\rangle d\mu _f\right. \\{} & {} \qquad \left. +\frac{n^2}{4}\int _\Omega |H|^2|\omega _i|^2d\mu _f -\frac{1}{4}\int _\Omega (2\Delta f+|df|^2)|\omega _i|^2d\mu _f\right) , \end{aligned}$$

for $\alpha \ge 2$. Here, $\omega _i$ represents the *i*-th eigenform of $\Delta _f$.

### Remark 1.4

In Theorem 1.1 and Theorem 1.3 the tensor $\mathfrak {B}^{[p]}$ appears in the estimates. It is possible to express this tensor in terms of extrinsic geometric data via Eq. (4.5) but as this would further complicate the presentation of the eigenvalue estimates we do not make use of this option.

In order to eliminate the dependence on $\omega _i$ in the statements of Theorem 1.3, we take the infimum over $\Omega$ of the smallest eigenvalue of the endomorphsim $\mathfrak {B}^{[p]}+T_f^{[p]}$ which we denote by $\delta _1$ to deduce that

### Corollary 1.5

Let $X:(M^n,g)\rightarrow (\mathbb {R}^{n+m}, \textrm{can})$ be an isometric immersion. For any $p\in \{0,\ldots ,n\}$, the eigenvalues of the drifting Hodge Laplacian $\Delta _f$ acting on *p*-forms on a domain $\Omega$ of *M* with Dirichlet boundary conditions satisfy for any $k\ge 1$

$$\begin{aligned} \sum _{i=1}^k(\lambda _{k+1,p,f}-\lambda _{i,p,f})^\alpha \le \frac{4}{n}\sum _{i=1}^k(\lambda _{k+1,p,f}-\lambda _{i,p,f})^{\alpha -1}\left( \lambda _{i,p,f}-\delta _1+\frac{1}{4}\delta _2\right) \end{aligned}$$

for $\alpha \le 2$. Also, we have

$$\begin{aligned} \sum _{i=1}^k(\lambda _{k+1,p,f}-\lambda _{i,p,f})^\alpha \le \frac{2\alpha }{n}\sum _{i=1}^k(\lambda _{k+1,p,f}-\lambda _{i,p,f})^{\alpha -1} \left( \lambda _{i,p,f}-\delta _1+\frac{1}{4}\delta _2\right) \end{aligned}$$

for $\alpha \ge 2$. Here, we set $\delta _2={\mathop {\textrm{sup}}\limits _{\Omega }} (n^2|H|^2-2\Delta f-|df|^2)$.

### Remark 1.6

1. Choosing $\alpha =2$ in the estimates of Corollary 1.5 generalizes the results obtained by Xia and Xu [39] for functions to the case of *p*-forms.
2. In the case of $\alpha =2$ and $f=\frac{|X|^2}{2}$ recursion formulas similar to the ones of Corollary 1.5 were established in [12].

Finally, we finish by establishing an estimate for the index of a compact *f*-minimal hypersurface of $\mathbb {R}^{n+1}$ as in [21, 37]. Recall that a *f*-minimal hypersurface *M* of the weighted manifold $(\mathbb {R}^{n+1},\textrm{can}, e^{-f}dv_g)$ is a hypersurface such that the *f*-mean curvature vanishes,

$$\begin{aligned} nH_f:=nH+\frac{\partial f}{\partial \nu }=0, \end{aligned}$$

where *H* is the mean curvature. The *stability operator* of *M*, also called *Jacobi operator*, is then defined for any smooth function *u* on *M* by

$$\begin{aligned} L_f(u):=\Delta _f u-(\textrm{Ric}_f^{\mathbb {R}^{n+1}}(\nu ,\nu )+|\textbf{II}|^2)u, \end{aligned}$$

where $\textrm{Ric}_f^{\mathbb {R}^{n+1}}$ denotes the Bakry-Emery Ricci tensor. On an arbitrary Riemannian manifold (*N*, *h*), the Bakry-Emery Ricci tensor (or the $\infty$-Bakry-Emery Ricci tensor) is defined as follows

$$\begin{aligned} \text {Ric}^N_f=\text {Ric}^N+\text {Hess}^N f, \end{aligned}$$

where $\textrm{Hess}^{N} f$ is the Hessian of *f* on *N*. In the case of $N=(\mathbb {R}^n,\mathrm can)$ we simply have that $\textrm{Ric}_f^{\mathbb {R}^{n+1}}=\textrm{Hess}^{\mathbb {R}^{n+1}} f$ and the stability operator acquires the form

$$\begin{aligned} L_f(u):=\Delta _f u-(\textrm{Hess}^{\mathbb {R}^{n+1}} f(\nu ,\nu )+|\textbf{II}|^2)u. \end{aligned}$$

The *f*-index of *M* is the number of negative eigenvalues of the Jacobi operator $L_f$. In order to estimate this number, we will establish an upper bound for the eigenvalues $\big (\lambda _k(L_f)\big )_k$ of the Jacobi operator that depends on the eigenvalues of the drifting Hodge Laplacian $\Delta _f$ on *p*-forms. This will generalize the result obtained in [21, Theorem A] when $p=1$.

### Theorem 1.7

Let $(M^n,g)$ be a compact hypersurface of the weighted manifold $(\mathbb {R}^{n+1}, \textrm{can}, d\mu _f=e^{-f}dv_g)$. Assume that *M* is *f*-minimal and that $\textrm{Ric}_f^{\mathbb {R}^{n+1}}=\textrm{Hess}^{\mathbb {R}^{n+1}} f\ge a>0$. We denote by $k_1,\ldots ,k_n$ the principal curvatures of the hypersurface. Then, for all $l\ge 1$ and $1\le p\le n$, we have that

$$\begin{aligned} \lambda _l(L_f)\le \lambda _{d(l),p,f}-(p+1)a+\gamma _M p(p-1), \end{aligned}$$

where $$\gamma _M=\textrm{sup}\{k_ik_j: i\ne j\}$$ and

$$\begin{aligned} d(l)=\begin{pmatrix}n+1\\ p+1\end{pmatrix}(l-1)+1. \end{aligned}$$

Now, if we set

$$\begin{aligned} \beta =\sharp \{\text {eigenvalues on}\,\, p\text {-forms of}\, \Delta _f\, \text {which are less than}\, (p+1)a-\gamma _M p(p-1)\}, \end{aligned}$$

we obtain the immediate two corollaries

### Corollary 1.8

Let $(M^n,g)$ be a compact hypersurface of the weighted manifold $(\mathbb {R}^{n+1}, \textrm{can}, d\mu _f=e^{-f}dv_g)$. Assume that *M* is *f*-minimal and that $\textrm{Ric}_f^{\mathbb {R}^{n+1}}=\textrm{Hess}^{\mathbb {R}^{n+1}} f\ge a>0$. Then, for any $1\le p\le n$, we have that

$$\begin{aligned} \textrm{Ind}_f(M)\ge \frac{1}{\begin{pmatrix}n+1\\ p+1\end{pmatrix}}\beta . \end{aligned}$$

A particular case of Corollary 1.8 is when *M* is self-shrinker. Recall that a self-shrinker manifold is a compact and connected submanifold of the Euclidean space $\mathbb {R}^{n+m}$ satisfying the equation $X^\perp =-nH$. Here $X=(x_1,x_2,\ldots ,x_{n+m})$ are the components of the immersion into $\mathbb {R}^{n+m}$. By taking the weight $f=\frac{|X|^2}{2}$, one can easily check that any self-shrinker hypersurface is automatically *f*-minimal and that $\textrm{Hess}^{\mathbb {R}^{n+1}} f=\textrm{can}$. Therefore,

### Corollary 1.9

Let $(M^n,g)\rightarrow \mathbb {R}^{n+1}$ be a compact self-shrinker such that $p(p-1)\gamma _M\le p+1$ for some $1\le p\le n$. Then, we have

$$\begin{aligned} \textrm{Ind}_f(M)\ge \frac{1}{\begin{pmatrix}n+1\\ p+1\end{pmatrix}}b_p(M)+n+1, \end{aligned}$$

for the weight $f=\frac{|X|^2}{2}$.

### Remark 1.10

We would like to point out that Theorem 1.7 allows to obtain a statement on the stability of *f*-minimal hypersurfaces in terms of its *p*-th Betti number which was only known for $p=1$ so far. However, the drawback is the fact that the stability estimate also depends on the curvature of the hypersurface which is not the case for $p=1$.

This article is organized as follows: In Sect. 2 we present a number of general features on Dirac and Laplace type operators on weighted manifolds. Section 3 provides the proofs of the main results while Sect. 4 is devoted to a number of geometric applications. Throughout this article, we will use the geometers sign convention for the Laplacian.

When finalizing this manuscript, the article “Spinors and mass on weighted manifolds” [5] appeared, where most of the results presented in Subsection 2.1 have been obtained independently from our calculations.

## Dirac and Laplace Type Operators on Weighted Manifolds

### The Dirac Operator on Weighted Manifolds

In this section, we study the question of defining the fundamental Dirac operator on a weighted Riemannian spin manifold. In the famous article of Perelman [33, Remark 1.3] some results in this direction were already presented.

In particular, this will allow to get a new vanishing result on the kernel of the Dirac operator based on the scalar curvature of weighted manifolds, see also [33, Remark 1.3].

In order to approach this question, we first recall some basic facts on spin manifolds [23]. Let $(M^n,g)$ be a Riemannian spin manifold of dimension *n*. The spinor bundle $\Sigma M$ is a vector bundle over the manifold *M* that is equipped with a metric connection $\nabla$ and a Hermitian scalar product $\langle \cdot ,\cdot \rangle$. On this bundle we have the Clifford multiplication of spinors with tangent vectors which we denote by the symbol $``\cdot$”. Remember that Clifford multiplication is compatible with the connection on the spinor bundle. Moreover, it is skew-symmetric in the sense

$$\begin{aligned} \langle Y\cdot \psi ,\varphi \rangle =-\langle \psi ,Y\cdot \varphi \rangle \end{aligned}$$

for all $Y\in TM$ and $\psi ,\varphi \in \Gamma (\Sigma M)$. The fundamental Dirac operator $D:\Gamma (\Sigma M)\rightarrow \Gamma (\Sigma M)$ acting on spinors is defined by

$$\begin{aligned} D:=\sum _{i=1}^ne_i\cdot \nabla _{e_i}, \end{aligned}$$

where $\{e_i\}_{i=1,\ldots ,n}$ is an orthonormal basis of *TM*. The Dirac operator is an elliptic, first order differential operator which is self-adjoint with respect to the $L^2$-norm (when *M* is compact). Therefore, it admits a sequence of eigenvalues of finite multiplicities. Also, its square is related to the Laplacian through the so-called *Schrödinger-Lichnerowicz formula* which is given by [23]

2.1 $$\begin{aligned} D^2=\nabla ^*\nabla +\frac{1}{4}\text {Scal}^M, \end{aligned}$$

where $\nabla ^*\nabla =-\sum _{i=1}^n \nabla _{e_i}\nabla _{e_i}+\sum _{i=1}^n \nabla _{\nabla _{e_i} e_i}$ and $\textrm{Scal}^M$ is the scalar curvature of the manifold *M*. Let us now consider a smooth real valued function *f* on *M* and the weighted measure $d\mu _f:=e^{-f}dv_g$. In order to define the Dirac operator on a compact weighted manifold, we use the following variational principle. The standard Dirac action with a weighted measure is

2.2 $$\begin{aligned} E(\psi ):=\int _M\langle \psi ,D\psi \rangle d\mu _f, \end{aligned}$$

where $$\psi \in \Gamma (\Sigma M)$$.

#### Proposition 2.1

The critical points of (2.2) are characterized by the equation

2.3 $$\begin{aligned} D\psi -\frac{1}{2}df\cdot \psi =0. \end{aligned}$$

#### Proof

We consider a variation of the spinor $\psi$ defined as $\psi _t:(-\varepsilon ,\varepsilon )\times M\rightarrow \Sigma M$ satisfying $\frac{\nabla \psi _t}{\partial t}\big |_{t=0}=\varphi$ and we calculate

$$\begin{aligned} \frac{d}{dt}\big |_{t=0}E(\psi _t)&=\int _M(\langle \varphi ,D\psi \rangle +\langle \psi ,D\varphi \rangle ) d\mu _f \\&=\int _M\big (\langle \varphi ,D\psi \rangle +\langle D\psi ,\varphi \rangle -\langle df\cdot \psi ,\varphi \rangle \big ) d\mu _f\\&=\int _M\big (\text {Re}\langle (2D-df\cdot )\psi ,\varphi \rangle d\mu _f. \end{aligned}$$

Therefore, the critical points of the energy *E* are exactly solutions of Eq. (2.2). This completes the proof. $\square$

Motivated by the above calculation, we define

#### Definition 2.2

The fundamental Dirac operator on a Bakry-Emery manifold $(M^n,g, d\mu _f)$ is given by

2.4 $$\begin{aligned} D_f:=D-\frac{1}{2}df\cdot \end{aligned}$$

#### Proposition 2.3

The operator $D_f$ is elliptic. Moreover, if *M* is compact, it is self-adjoint with respect to the $L^2$-norm.

#### Proof

It is straightforward to check that *D* and $D_f$ have the same principal symbol, hence $D_f$ is clearly elliptic. To prove the second part, we choose two spinors $\psi ,\varphi$ and calculate

$$\begin{aligned} \int _M\langle D_f\psi ,\varphi \rangle e^{-f} dv_g&= \int _M\langle D\psi ,\varphi \rangle e^{-f} dv_g -\frac{1}{2}\int _M\langle df\cdot \psi ,\varphi \rangle e^{-f} dv_g\\&=\int _M\langle \psi ,D(e^{-f}\varphi )\rangle dv_g -\frac{1}{2}\int _M\langle df\cdot \psi ,\varphi \rangle e^{-f} dv_g\\&=\int _M\langle \psi ,D\varphi \rangle e^{-f} dv_g -\int _M\langle \psi ,df\cdot \varphi \rangle e^{-f} dv_g\\&\quad +\frac{1}{2}\int _M\langle \psi , df\cdot \varphi \rangle e^{-f} dv_g\\&=\int _M\Bigg \langle \psi ,D\varphi -\frac{1}{2}df\cdot \varphi \Bigg \rangle e^{-f} dv_g\\&=\int _M\langle \psi ,D_f\varphi \rangle e^{-f} dv_g. \end{aligned}$$

The proof is complete. $\square$

#### Remark 2.4

It is straightforward to check that (2.2) is invariant under conformal transformations of the metric on *M*. Hence, we cannot expect that we can relate the properties of *D* and $D_f$ by conformally transforming the metric on *M*.

However, the spectral properties of $D_f$ are essentially the same as the ones of the standard Dirac operator *D* as is shown by the following proposition.

#### Proposition 2.5

On a Bakry-Emery manifold $(M^n,g, d\mu _f)$, we have

$$\begin{aligned} D_f(e^{f/2})=e^{f/2} D. \end{aligned}$$

In particular, if $\psi$ is an eigenspinor of *D* associated with the eigenvalue $\lambda$, then $e^{f/2} \psi$ is an eigenspinor of $D_f$ associated with the same eigenvalue $\lambda$.

#### Proof

By a straightforward calculation, we have for any spinor field $\psi$

$$\begin{aligned} D_f(e^{f/2}\psi )= & {} D(e^{f/2}\psi )-\frac{1}{2}e^{f/2}df\cdot \psi \\= & {} e^{f/2}D\psi +\frac{1}{2}e^{f/2}df\cdot \psi -\frac{1}{2}e^{f/2}df\cdot \psi \\= & {} e^{f/2} D\psi . \end{aligned}$$

$$\square $$

In order to obtain a corresponding *Schrödinger-Lichnerowicz formula* for $D_f$ we make the following observation: The $L^2$-adjoint of $\nabla$ with respect to the weighted measure $e^{-f}dv_g$ will be denoted by $\nabla ^*_f$ and is given by the following expression

$$\begin{aligned} \nabla ^*_f=\nabla ^*+\nabla _{df}. \end{aligned}$$

Hence, we have

#### Proposition 2.6

The square $D_f^2$ satisfies the following formula:

2.5 $$\begin{aligned} D_f^2=\nabla _f^*\nabla +\frac{1}{4}\text {Scal}^M -\frac{1}{2}\Delta f-\frac{1}{4} |df|^2. \end{aligned}$$

#### Proof

A direct calculation shows

$$\begin{aligned} D_f^2= & {} \left( D-\frac{1}{2} df\cdot \right) \left( D-\frac{1}{2} df\cdot \right) \\= & {} D^2-\frac{1}{2}D(df\cdot )-\frac{1}{2}df\cdot D+\frac{1}{4} df\cdot df\cdot \\= & {} D^2-\frac{1}{2}(\delta df-df\cdot D-2\nabla _{df})-\frac{1}{2}df\cdot D+\frac{1}{4} df\cdot df\cdot \\= & {} D^2-\frac{1}{2}\Delta f+\nabla _{df}-\frac{1}{4} |df|^2. \end{aligned}$$

Combining with (2.1) and using the definition of $\nabla ^*_f$ completes the proof. $\square$

As in the case of the fundamental Dirac operator on Riemannian spin manifolds [24], we deduce the following vanishing result

#### Corollary 2.7

Let $(M,g,d\mu _f)$ be a closed Bakry-Emery manifold. If $\text {Scal}^M>2\Delta f+|df|^2$, then $\ker D=0$.

#### Proof

This follows directly from Proposition 2.6 and the invariance of the spectrum of the Dirac operator in Proposition 2.5. $\square$

#### Remark 2.8

Suppose that (*M*, *g*) is a closed Riemannian surface. If we integrate the condition $\text {Scal}^M>2\Delta f+|df|^2$ with respect to the standard Riemannian measure $dv_g$, we get

$$\begin{aligned} 2\pi \chi (M)-\int _M|df|^2 dv_g>0, \end{aligned}$$

where $\chi (M)$ represents the Euler characteristic of the surface. Hence, this inequality can only be satisfied on a surface of positive Euler characteristic.

### The Bochner-Laplacian on Weighted Manifolds

In this section, we will recall the Hodge Laplacian on weighted manifolds and define a twisted Laplacian motivated from the expression of the Dirac operator in the previous section. This will also allow to get a new vanishing result on the cohomology groups of the manifold.

For this, let $(M^n,g,d\mu _f=e^{-f}dv_g)$ be a Riemannian Bakry-Emery manifold. We denote by *d* the exterior differential and by $\delta$ the codifferential. The weighted codifferential is defined by $\delta _f:=\delta +df\lrcorner$ where we identify here (and in all the paper) vectors with one-forms through the musical isomorphism. The weighted codifferential is the $L^2$-formal adjoint of *d* with respect to the measure $d\mu _f$, when *M* is compact. By a straightforward computation, one shows that

$$\begin{aligned} \delta _f^2=\delta (\delta +df\lrcorner )+df\lrcorner (\delta +df\lrcorner )=\delta (df\lrcorner )+df\lrcorner \delta =0, \end{aligned}$$

where we use the fact $\delta (df\lrcorner )=-df\lrcorner \delta$, since $\textrm{Hess}^Mf=\nabla df$ is a symmetric endomorphism. The drifting Hodge Laplacian on differential forms is then defined as

$$\begin{aligned} \Delta _f:=d\delta _f+\delta _f d. \end{aligned}$$

Clearly, the drifting Hodge Laplacian commutes with *d* and $\delta _f$ and, therefore, it preserves the spaces of exact and weighted coexact forms. Moreover, this operator is elliptic and, when *M* is compact, it is self-adjoint with respect to the weighted measure $d\mu _f$. Therefore, as for the ordinary Hodge Laplacian on compact manifolds, the drifting Hodge Laplacian restricted to differential *p*-forms ($1\le p\le n$) has a spectrum that consists of a nondecreasing, unbounded sequence of eigenvalues with finite multiplicities, that is

$$\begin{aligned} \text {Spec}(\Delta _f)=\{0\le \lambda _{1,p,f}\le \lambda _{2,p,f}\le \ldots \}. \end{aligned}$$

The eigenvalue 0 corresponds to the space of *f*-harmonic forms, that is differential forms $\omega$ satisfying $d\omega =0$ and $\delta _f\omega =0$. Notice that, by standard elliptic theory, we have an isomorphism [25, Formula 2.13],

$$\begin{aligned} H^p(M)\simeq \{\omega \in \Omega ^p(M)|\,\, d\omega =0,\, \delta _f\omega =0\}, \end{aligned}$$

meaning that the *f*-Betti numbers do not depend on *f*. Now it is shown in [32], that the drifting Hodge Laplacian $\Delta _f$ has a corresponding Bochner-Weitzenböck formula which is

2.6 $$\begin{aligned} \Delta _f=\nabla _f^*\nabla +\mathfrak {B}^{[p]}+T_f^{[p]} \end{aligned}$$

where $\mathfrak {B}^{[p]}=\sum _{i,j=1}^ne_j^*\wedge e_i\lrcorner R^M(e_i,e_j)$ is the Bochner operator that appears in the Bochner-Weitzenböck formula for $\Delta =d\delta +\delta d$. Here, $R^M(X,Y):=[\nabla _X,\nabla _Y]-\nabla _{[X,Y]}$ is the curvature tensor operator of *M* for $X,Y\in TM$ and $T_f^{[p]}$ is the self-adjoint endomorphism of $\Lambda ^p(M)$ given by

2.7 $$\begin{aligned} (T_f^{[p]}\omega )(X_1,\ldots ,X_p)=\sum _{k=1}^p\omega (X_1,\ldots ,\nabla ^2f(X_k),\ldots ,X_p) \end{aligned}$$

for all $X_1,\ldots , X_p\in TM$ and $\{e_1,\ldots ,e_n\}$ is a local orthonormal frame of *TM*. The tensor $\mathfrak {B}^{[p]}+T_f^{[p]}$ is called the *p-Ricci tensor* and is denoted by $\textrm{Ric}^{(p)}_f$ (see [34]). For $p=1$, the *p*-Ricci tensor is just $\textrm{Ric}^{(1)}_f=\textrm{Ric}^M+\textrm{Hess}^M f$ which is the $\infty$-Bakry-Emery Ricci tensor. Also, for any $N\in ]-\infty ,0[\cup ]n-p+1,\infty [$, we let

$$\begin{aligned} \textrm{Ric}^{(p)}_{N,f}:= \textrm{Ric}^{(p)}_{f}-\frac{1}{N-(n-p+1)}(df\wedge (df\lrcorner )) \end{aligned}$$

which corresponds to the so-called *N*-Bakry-Emery Ricci tensor for $p=1$. It is now a well-known fact that the Bochner-Weitzenböck formula gives rise to the Gallot-Meyer estimate on manifolds having a lower bound on the Bochner operator $\mathfrak {B}^{[p]}$ [15]. In the following, we will adapt this technique to give an estimate for the eigenvalues of the drifting Hodge Laplacian on a Bakry-Emery manifold having a lower bound on $\textrm{Ric}^{(p)}_{N,f}$. For this, we denote by $\lambda '_{1,p,f}$ the first positive eigenvalue of $\Delta _f$ restricted to exact *p*-forms. Then, we have

#### Proposition 2.9

Let $(M^n,g,d\mu _f)$ be a compact Bakry-Emery manifold. If $\textrm{Ric}^{(p)}_{N,f}\ge p(n-p)\gamma$ for some $\gamma >0$, then the first eigenvalue $\lambda '_{1,p,f}$ satisfies the estimate

$$\begin{aligned} \lambda '_{1,p,f}\ge p(n-p)\gamma \frac{N}{N-1}. \end{aligned}$$

#### Proof

Let $\omega$ be any exact *p*-eigenform. Applying the Bochner-Weitzenböck formula (2.6) yields

$$\begin{aligned} \lambda '_{1,p,f}\int _M|\omega |^2d\mu _f=\int _M|\nabla \omega |^2d\mu _f+\int _M\langle (\mathfrak {B}^{[p]}+T_f^{[p]})\omega ,\omega \rangle d\mu _f. \end{aligned}$$

Now, using that $|\nabla \omega |^2\ge \frac{1}{n-p+1}|\delta \omega |^2$ as $\omega$ is closed [15], we have that

$$\begin{aligned} |\nabla \omega |^2\ge & {} \frac{1}{n-p+1}|\delta _f\omega -df\lrcorner \omega |^2\\\ge & {} \frac{1}{n-p+1}(|\delta _f\omega |-|df\lrcorner \omega |)^2\\\ge & {} \frac{1}{N}|\delta _f\omega |^2-\frac{1}{N-(n-p+1)}|df\lrcorner \omega |^2. \end{aligned}$$

Here, we use the inequality $\frac{(p+q)^2}{s}\ge \frac{p^2}{N}-\frac{q^2}{N-s}$ for all *N* such that we have $N(N-s)>0$. Therefore, by integrating over *M* and using the fact $\lambda '_{1,p,f}\int _M|\omega |^2d\mu _f=\int _M|\delta _f\omega |^2d\mu _f$, we get the estimate. $\square$

In the last part of this section, we will define a new drifting Hodge Laplacian acting on differential *p*-forms that will allow us to get a new vanishing result on the cohomology groups. For this, recall that the weighted Dirac operator defined in the previous section is $D_f=\sum _{i=1}^n e_i\cdot \nabla _{e_i}-\frac{1}{2}df\cdot$. When restricted to differential forms, the operator $D_f$ can be written as

$$\begin{aligned} D_f=\sum _{i=1}^n e_i\wedge \nabla _{e_i}-e_i\lrcorner \nabla _{e_i}-\frac{1}{2}df\wedge +\frac{1}{2}df\lrcorner =\widetilde{d}_f+\widetilde{\delta }_f, \end{aligned}$$

where $\widetilde{d}_f:=d-\frac{1}{2}df\wedge$ and $\widetilde{\delta }_f:=\delta +\frac{1}{2}df\lrcorner$. Here, we use the fact that $X\cdot \omega =X\wedge \omega -X\lrcorner \omega$ for any vector field *X* and a differential form $\omega$. It is not difficult to check that $\widetilde{d}_f^2=\widetilde{\delta }_f^2=0$ and that $\widetilde{\delta }_f=\delta _f-\frac{1}{2}df\lrcorner$ is the $L^2$-adjoint of $\widetilde{d}_f$ with respect to the measure $d\mu _f=e^{-f}dv_g$. Also the square of the Dirac operator gives rise to the twisted Laplacian

$$\begin{aligned} D_f^2=\widetilde{ \Delta }_f:=\widetilde{d}_f\widetilde{\delta }_f+\widetilde{\delta }_f\widetilde{d}_f \end{aligned}$$

that has the same spectrum as $\Delta$ by Proposition 2.5. Now, we establish a Bochner-Weitzenböck formula for $\widetilde{\Delta }_f$ in order to get a new vanishing result for the cohomology groups.

#### Proposition 2.10

Let $(M,g,d\mu _f)$ be a Bakry-Emery manifold. Then, we have the Bochner-Weitzenböck formula for the twisted drifting Hodge Laplacian

2.8 $$\begin{aligned} \widetilde{ \Delta }_f=\nabla _f^*\nabla +\mathfrak {B}^{[p]}-\frac{1}{2}\Delta f-\frac{1}{4}|df|^2. \end{aligned}$$

In particular, if *M* is compact and $\mathfrak {B}^{[p]}>\frac{1}{2}\Delta f+\frac{1}{4}|df|^2$ for some *p*, then $H^p(M)=0$.

#### Proof

We compute

$$\begin{aligned} \widetilde{ \Delta }_f= & {} \widetilde{d}_f\widetilde{\delta }_f+\widetilde{\delta }_f\widetilde{d}_f\\= & {} \left( d-\frac{1}{2}df\wedge \right) \left( \delta _f-\frac{1}{2} df\lrcorner \right) +\left( \delta _f-\frac{1}{2} df\lrcorner \right) \left( d-\frac{1}{2}df\wedge \right) \\= & {} \Delta _f-\frac{1}{2}d(df\lrcorner )-\frac{1}{2}df\wedge \delta _f+\frac{1}{4}df\wedge (df\lrcorner )-\frac{1}{2}\delta _f(df\wedge )-\frac{1}{2}df\lrcorner d+\frac{1}{4}df\lrcorner (df\wedge )\\= & {} \Delta _f-\frac{1}{2}\mathcal {L}_{df}-\frac{1}{2}df\wedge \delta _f-\frac{1}{2}\delta _f(df\wedge )+\frac{1}{4}|df|^2\\= & {} \Delta _f-\frac{1}{2}\nabla _{df}-\frac{1}{2}T^{[p]}_f-\frac{1}{2}df\wedge \delta _f-\frac{1}{2}\delta _f(df\wedge )+\frac{1}{4}|df|^2. \end{aligned}$$

In the last equality, we used the identity [36, Lem. 2.1]

$$\begin{aligned} \mathcal {L}_{df}=\nabla _{df}+T_f^{[p]}. \end{aligned}$$

Now, an easy computation shows that

$$\begin{aligned} \delta _f(df\wedge )=\Delta f+\sum _{i=1}^n\nabla _{e_i}df\wedge (e_i\lrcorner )-\nabla _{df}+|df|^2-df\wedge \delta _f. \end{aligned}$$

Using the fact that $T_f^{[p]}=\sum _{i=1}^n\nabla _{e_i}df\wedge (e_i\lrcorner )$ which can be proven by a straightforward computation, we get after using Eq. (2.6) and replacing the last equality,

$$\begin{aligned} \widetilde{ \Delta }_f= & {} \nabla _f^*\nabla +\mathfrak {B}^{[p]}+T_f^{[p]}-\frac{1}{2}\nabla _{df}-\frac{1}{2}T^{[p]}_f-\frac{1}{2}df\wedge \delta _f \\{} & {} -\frac{1}{2}\left( \Delta f+T_f^{[p]}-\nabla _{df}+|df|^2-df\wedge \delta _f\right) +\frac{1}{4}|df|^2\\= & {} \nabla _f^*\nabla +\mathfrak {B}^{[p]}-\frac{1}{2}\Delta f-\frac{1}{4}|df|^2. \end{aligned}$$

Note that (2.8) has the same structure as the corresponding Schrödinger-Lichnerowicz formula for the Dirac operator on weighted manifolds (2.5). The vanishing result on the cohomology is obtained by just applying (2.8) to a $\widetilde{ \Delta }_f$-harmonic form and integrating over *M*. Finally, the fact that the set of $\widetilde{ \Delta }_f$-harmonic form is isomorphic to $H^p(M)$ allows to finish the proof. $\square$

## Proofs of the Main Results

In this section, we provide the proofs of the main results.

### Proof of Theorem 1.1

We start by proving the first part of the theorem. We assume that $(M^n,g, d\mu _f=e^{-f}dv_g)$ is a compact Bakry-Emery manifold that is isometrically immersed into $\mathbb {R}^{n+m}$. For $p=1,\ldots ,n$, we let $\lambda _{1,p,f}$ the first nonegative eigenvalue of $\Delta _f$ on *p*-forms and $\lambda '_{1,p,f}$ the first positive eigenvalue of $\Delta _f$ on exact *p*-forms. For every $i=1,\ldots ,n+m$, the parallel unit vector field $\partial x_i$ decomposes into $\partial x_i=(\partial x_i)^T+(\partial x_i)^\perp$ with $(\partial x_i)^T=d(x_i\circ \iota )$ where $\iota$ is the isometric immersion. Now, take any *p*-eigenform $\omega$ of $\Delta _f$ and consider the $(p-1)$-form $\phi _i:=(\partial x_i)^T\lrcorner \omega$ for each $i=1,\ldots , n+m$. By the Rayleigh min-max principle, we have that

3.1 $$\begin{aligned} \lambda _{1,p-1,f}\sum _{i=1}^{n+m}\int _M|\phi _i|^2d\mu _f\le \sum _{i=1}^{n+m}\int _M(|d\phi _i|^2 +|\delta _f\phi _i|^2)d\mu _f. \end{aligned}$$

In the following, we will adapt some computations done in [16] to the context of the drifting Hodge Laplacian (see also [1], [8, Thm. 5.8] for a similar computation). Denoting by $\{e_1,\ldots , e_n\}$ a local orthonormal frame of *TM* we find that

3.2 $$\begin{aligned} \sum _{i=1}^{n+m}|\phi _i|^2=\sum _{s,t=1}^n\underbrace{\sum _{i=1}^{n+m}g((\partial x_i)^T,e_s)g((\partial x_i)^T,e_t)}_{\delta _{st}}\langle e_s\lrcorner \omega ,e_t\lrcorner \omega \rangle =\sum _{s=1}^n |e_s\lrcorner \omega |^2=p|\omega |^2. \end{aligned}$$

Here, we use the fact that $\alpha =\frac{1}{p}\sum _{s=1}^n e_s^*\wedge e_s\lrcorner \alpha$, for any *p*-form $\alpha$. Moreover, by using the Cartan formula and [16, Eq. (4.3)], we write

$$\begin{aligned} d\phi _i=\mathcal {L}_{(\partial x_i)^T}\omega -(\partial x_i)^T\lrcorner d\omega =\nabla _{(\partial x_i)^T}\omega +\textbf{II}_{(\partial x_i)^\perp }^{[p]}\omega -(\partial x_i)^T\lrcorner d\omega , \end{aligned}$$

where ${\textbf{II}}_{Z}^{[p]}$ is the canonical extension of the second fundamental form $\textbf{II}$ of the immersion to differential *p*-forms in the normal direction *Z*. More precisely, if we write $\langle {\textbf{II}}_Z(X),Y\rangle =\langle {\textbf{II}}(X,Y),Z\rangle$ for any tangent vector fields $X,Y\in TM$ and $Z\in T^\perp M$, we define

3.3 $$\begin{aligned} ({\textbf{II}}_Z^{[p]}\omega )(X_1,\ldots , X_p):=\sum _{i=1}^p \omega (X_1,\ldots , {\textbf{II}}_Z(X_i),\ldots , X_p) \end{aligned}$$

for any differential *p*-form $\omega$ on *M* and $X_1,\ldots ,X_p\in TM$. Now, as we did in (3.1), we decompose $(\partial x_i)^T$ and $(\partial x_i)^\perp$ in the frames $\{e_1,\ldots ,e_n\}$ and $\{\nu _1,\ldots ,\nu _m\}$ to compute the norm square of $d\phi _i$, we deduce after summing over *i* that

3.4 $$\begin{aligned} \sum _{i=1}^{n+m}|d\phi _i|^2= & {} |\nabla \omega |^2+\sum _{s=1}^m|{\textbf{II}}^{[p]}_{\nu _s}\omega |^2+(p+1)|d\omega |^2-2\sum _{i=1}^n \langle \nabla _{e_i}\omega ,e_i\lrcorner d\omega \rangle \nonumber \\= & {} |\nabla \omega |^2+\sum _{s=1}^m|{\textbf{II}}^{[p]}_{\nu _s}\omega |^2+(p-1)|d\omega |^2. \end{aligned}$$

Here, $\{\nu _1,\ldots ,\nu _m\}$ is a local orthonormal frame of $T^\perp M$. In the above computation, we use the fact that all cross terms involving $(\partial x_i)^T$ and $(\partial x_i)^\perp$ are zero. By writing $\delta _f\phi _i=\delta \phi _i+df\lrcorner \phi _i$ with $\delta \phi _i=-(\partial x_i)^T\lrcorner \delta \omega$ which is a consequence from the fact that $\nabla (\partial x_i)^T=\textrm{Hess}^M(x_i\circ \iota )$ is a symmetric endomorphism, we get that

3.5 $$\begin{aligned} \sum _{i=1}^{n+m}|\delta _f\phi _i|^2= & {} (p-1)|\delta \omega |^2+(p-1)|df\lrcorner \omega |^2 -2\sum _{i=1}^{n+m}\langle (\partial x_i)^T\lrcorner \delta \omega , df\lrcorner (\partial x_i)^T\lrcorner \omega \rangle \nonumber \\= & {} (p-1)|\delta \omega |^2+(p-1)|df\lrcorner \omega |^2 -2\sum _{i=1}^{n}\langle e_i\lrcorner \delta \omega , df\lrcorner e_i\lrcorner \omega \rangle \nonumber \\= & {} (p-1)|\delta \omega |^2+(p-1)|df\lrcorner \omega |^2+2(p-1)\langle \delta \omega ,df\lrcorner \omega \rangle \nonumber \\= & {} (p-1)|\delta _f\omega |^2. \end{aligned}$$

Replacing Equalities (3.2), (3.4) and (3.5) into Inequality (3.1), we deduce after using the Bochner-Weitzenböck formula (2.6) that

$$\begin{aligned} p\lambda _{1,p-1,f}\int _M|\omega |^2d\mu _f\le & {} \int _M(|\nabla \omega |^2+\sum _{s=1}^m|{\textbf{II}}^{[p]}_{\nu _s}\omega |^2+(p-1)|d\omega |^2+(p-1)|\delta _f\omega |^2)d\mu _f\\= & {} \int _M(\langle \Delta _f\omega ,\omega |\Bigg \rangle -\Bigg \langle (\mathfrak {B}^{[p]}+T_f^{[p]})\omega ,\omega \Bigg \rangle +\sum _{s=1}^m|{\textbf{II}}^{[p]}_{\nu _s}\omega |^2)d\mu _f \\{} & {} +(p-1)\lambda _{1,p,f}\int _M|\omega |^2d\mu _f\\= & {} p\lambda _{1,p,f}\int _M|\omega |^2d\mu _f+\int _M\Bigg \langle \left( \sum _{s=1}^m(\mathbf{\textbf{II}}^{[p]}_{\nu _s})^2-\mathfrak {B}^{[p]}\right. \\{} & {} \left. -T_f^{[p]}\right) \omega ,\omega \Bigg \rangle d\mu _f. \end{aligned}$$

This finishes the proof of the first part. To prove the second part of Theorem 1.1, we use the same technique as in [35] and adapt it to the case of the drifting Hodge Laplacian. We consider the exact *p*-form

$$\begin{aligned} (\partial x_{i_1})^T\wedge \ldots \wedge (\partial x_{i_p})^T, \end{aligned}$$

for $i_k=1,\ldots , n+m, k=1,\ldots ,p$. By applying the min-max principle and summing over $i_1,\ldots ,i_p$, we get

3.6 $$\begin{aligned}{} & {} \lambda '_{1,p,f}\sum _{i_1,\ldots ,i_p}\int _M |(\partial x_{i_1})^T\wedge \ldots \wedge (\partial x_{i_p})^T|^2 d\mu _f\nonumber \\{} & {} \quad \le \sum _{i_1,\ldots ,i_p}\int _M|\delta _f\left( (\partial x_{i_1})^T\wedge \ldots \wedge (\partial x_{i_p})^T\right) |^2 d\mu _f. \end{aligned}$$

Now, we manipulate both sums the same way as in [35]. The sum on the left hand side of (3.6) is equal to $$p!\begin{pmatrix}n\\ p\end{pmatrix}$$ (see [35, Lemma 2.1] for more details). To confirm this fact, we denote as usual by $\{e_1,\ldots ,e_n\}$ a local orthonormal frame of *TM* and calculate

$$\begin{aligned}&\sum _{i_1,\ldots ,i_p}|(\partial x_{i_1})^T\wedge \ldots \wedge (\partial x_{i_p})^T|^2 \\ {}&\quad =\sum _{i_1,\ldots ,i_p}\langle (\partial x_{i_1})^T\wedge \ldots \wedge (\partial x_{i_p})^T,(\partial x_{i_1})^T\wedge \ldots \wedge (\partial x_{i_p})^T\rangle \\ {}&\quad =\sum _{i_1,\ldots ,i_p,s,t}g((\partial x_{i_1})^T,e_s)g((\partial x_{i_1})^T,e_t)\\ {}&\qquad \times \langle e_s\wedge \ldots \wedge (\partial x_{i_p})^T,e_t\wedge \ldots \wedge (\partial x_{i_p})^T\rangle \\ {}&\quad =\sum _{i_2,\ldots ,i_p,s,t}\delta _{st}\langle e_s\wedge (\partial x_{i_2})^T\wedge \ldots \wedge (\partial x_{i_p})^T,e_t\wedge (\partial x_{i_2})^T\wedge \ldots \wedge (\partial x_{i_p})^T\rangle \\ {}&\quad =\sum _{i_2,\ldots ,i_p,s}|e_s\wedge (\partial x_{i_2})^T\wedge \ldots \wedge (\partial x_{i_p})^T|^2\\ {}&\quad = (n-p+1)\sum _{i_2,\ldots ,i_p}|(\partial x_{i_2})^T\wedge \ldots \wedge (\partial x_{i_p})^T|^2. \end{aligned}$$

In the last line, we use the fact that, for any *p*-form $\omega$, the equality $\sum _{s=1}^n |e_s\wedge \omega |^2=(n-p)|\omega |^2$ holds true. Hence, by induction, we deduce that

3.7 $$\begin{aligned} \sum _{i_1,\ldots ,i_p}|(\partial x_{i_1})^T\wedge \ldots \wedge (\partial x_{i_p})^T|^2=(n-p+1)(n-p+2)\ldots n=p! \begin{pmatrix}n\\ p\end{pmatrix}.\nonumber \\ \end{aligned}$$

Now, we aim to compute the sum on the right hand side of (3.6). To this end we recall some computations done in [35]. First, by [35, Eq. 3.3],

$$\begin{aligned}{} & {} \delta \left( (\partial x_{i_1})^T\wedge \ldots \wedge (\partial x_{i_p})^T\right) \\{} & {} \quad =\sum _{s=1}^p (-1)^{s+1} T^{[p-1]}_{(\partial x_{i_s})^\perp }\Bigg ((\partial x_{i_1})^T\wedge \ldots \wedge \widehat{(\partial x_{i_s})^T}\wedge \ldots \wedge (\partial x_{i_p})^T\Bigg ) \end{aligned}$$

where, on differential *q*-forms, the operator $T_{\nu }^{[q]}$ is defined as

$$\begin{aligned} T_{\nu }^{[q]}:={\textbf{II}}_{\nu }^{[q]}-n \langle H,\nu \rangle I \end{aligned}$$

for any normal vector field $\nu$. Here, ${\textbf{II}}_{\nu }^{[q]}$ is the canonical extension of the second fundamental form as defined previously in (3.3). For any basis of orthonormal vector fields $\{\nu _1,\ldots ,\nu _m\}\in T_xM^\perp$, we set

$$\begin{aligned} ||{\textbf{II}}^{[q]}||^2=\sum _{s=1}^m ||{\textbf{II}}_{\nu _s}^{[q]}||^2 \quad \text {and}\quad ||T^{[q]}||^2=\sum _{s=1}^m ||T_{\nu _s}^{[q]}||^2. \end{aligned}$$

It was shown in [35, p. 592] that

3.8 $$\begin{aligned} \sum _{i_1,\ldots ,i_p}|\delta \left( (\partial x_{i_1})^T\wedge \ldots \wedge (\partial x_{i_p})^T\right) |^2=p!||T^{[p-1]}||^2 \end{aligned}$$

and the latter can be computed explicitly in terms of $|{\textbf{II}}|^2$ and the scalar curvature of *M* through the formula (see [35, Lemma 2.5])

$$\begin{aligned} ||T^{[p-1]}||^2=\begin{pmatrix}n\\ p\end{pmatrix}\left( pn|H|^2-\frac{p(p-1)}{n(n-1)}\textrm{Scal}^M\right) . \end{aligned}$$

Therefore, we compute

3.9 $$\begin{aligned}&|\delta _f\left( (\partial x_{i_1})^T\wedge \ldots \wedge (\partial x_{i_p})^T\right) |^2\nonumber \\&\quad = |\delta \left( (\partial x_{i_1})^T\wedge \ldots \wedge (\partial x_{i_p})^T\right) |^2+|df\lrcorner \left( (\partial x_{i_1})^T\wedge \ldots \wedge (\partial x_{i_p})^T\right) |^2\nonumber \\&\qquad +2\Bigg \langle \delta \left( (\partial x_{i_1})^T\wedge \ldots \wedge (\partial x_{i_p})^T\right) , df\lrcorner \left( (\partial x_{i_1})^T\wedge \ldots \wedge (\partial x_{i_p})^T\right) \Bigg \rangle . \end{aligned}$$

First, we show that the mixed term in (3.9) vanishes. For this, we write

$$\begin{aligned}&\Bigg \langle \delta \left( (\partial x_{i_1})^T\wedge \ldots \wedge (\partial x_{i_p})^T\right) , df\lrcorner \left( (\partial x_{i_1})^T\wedge \ldots \wedge (\partial x_{i_p})^T\right) \Bigg \rangle \\&\quad =\sum _{s=1}^p (-1)^{s+1}\Bigg \langle T^{[p-1]}_{(\partial x_{i_s})^\perp }((\partial x_{i_1})^T\wedge \ldots \wedge \widehat{(\partial x_{i_s})^T}\wedge \ldots \wedge (\partial x_{i_p})^T), df\lrcorner \left( (\partial x_{i_1})^T\right. \\&\qquad \left. \wedge \ldots \wedge (\partial x_{i_p})^T\right) \Bigg \rangle \\&\quad = \sum _{s=1}^p (-1)^{s+1}g((\partial x_{i_s})^\perp ,\nu _\alpha ) g((\partial x_{i_s})^T,e_\beta ) \\&\qquad \times \Bigg \langle T^{[p-1]}_{\nu _\alpha }((\partial x_{i_1})^T\wedge \ldots \wedge \widehat{(\partial x_{i_s})^T}\wedge \ldots \wedge (\partial x_{i_p})^T), df\lrcorner \left( (\partial x_{i_1})^T\wedge \ldots \wedge e_\beta \right. \\&\qquad \left. \wedge \ldots \wedge (\partial x_{i_p})^T\right) \Bigg \rangle \\&\quad = \sum _{s=1}^p (-1)^{s+1}g(\partial x_{i_s},\nu _\alpha ) g(\partial x_{i_s},e_\beta ) \\&\qquad \times \Bigg \langle T^{[p-1]}_{\nu _\alpha }((\partial x_{i_1})^T\wedge \ldots \wedge \widehat{(\partial x_{i_s})^T}\wedge \ldots \wedge (\partial x_{i_p})^T), df\lrcorner \left( (\partial x_{i_1})^T\wedge \ldots \wedge e_\beta \right. \\&\qquad \left. \wedge \ldots \wedge (\partial x_{i_p})^T\right) \Bigg \rangle . \end{aligned}$$

Hence, summing over $i_1,\ldots , i_p$, we deduce that

$$\begin{aligned} \sum _{i_1,\ldots ,i_p}\langle \delta \left( (\partial x_{i_1})^T\wedge \ldots \wedge (\partial x_{i_p})^T\right) , df\lrcorner \left( (\partial x_{i_1})^T\wedge \ldots \wedge (\partial x_{i_p})^T\right) \rangle =0. \end{aligned}$$

Now, it remains to compute the sum over $i_1,\ldots , i_p$ of the second term in (3.9). We establish the following lemma:

### Lemma 3.1

For any vector field $X\in TM$, we have

$$\begin{aligned} \sum _{i_1,\ldots ,i_p} |X\lrcorner \left( (\partial x_{i_1})^T\wedge \ldots \wedge (\partial x_{i_p})^T\right) |^2=p!\begin{pmatrix}n-1\\ p-1\end{pmatrix}|X|^2. \end{aligned}$$

### Proof

Using the formula $X\lrcorner (\alpha \wedge \omega )=g(X,\alpha ^\sharp )\omega -\alpha \wedge X\lrcorner \omega$, which is valid for any one-form $\alpha$, we write

$$\begin{aligned} X\lrcorner \left( (\partial x_{i_1})^T\wedge \ldots \wedge (\partial x_{i_p})^T\right)&=g(X,(\partial x_{i_1})^T)(\partial x_{i_2})^T\wedge \ldots \wedge (\partial x_{i_p})^T \\&\quad -(\partial x_{i_1})^T\wedge \left( X\lrcorner ((\partial x_{i_2})^T\ldots \wedge (\partial x_{i_p})^T)\right) . \end{aligned}$$

Taking the norm and summing over $i_1,\ldots , i_p$, we get that

$$\begin{aligned}&\sum _{i_1,\ldots ,i_p} |X\lrcorner \left( (\partial x_{i_1})^T\wedge \ldots \wedge (\partial x_{i_p})^T\right) |^2\\&\quad =\sum _{i_1,\ldots ,i_p} g(X,(\partial x_{i_1})^T)^2|(\partial x_{i_2})^T\wedge \ldots \wedge (\partial x_{i_p})^T|^2\\&\qquad +\sum _{i_1,\ldots ,i_p}|(\partial x_{i_1})^T\wedge \left( X\lrcorner ((\partial x_{i_2})^T\wedge \ldots \wedge (\partial x_{i_p})^T)\right) |^2\\&\qquad -2\sum _{i_1,\ldots ,i_p}g(X,(\partial x_{i_1})^T)\Bigg \langle (\partial x_{i_1})^T\lrcorner \left( (\partial x_{i_2})^T\wedge \ldots \wedge (\partial x_{i_p})^T\right) , X\lrcorner ((\partial x_{i_2})^T\\&\qquad \wedge \ldots \wedge (\partial x_{i_p})^T)\Bigg \rangle . \end{aligned}$$

Using the fact that $\sum _{i=1}^{n+m} g(X,(\partial x_i)^T)^2=|X|^2$ and Eq. (3.7), the first sum in the above equation is equal to $$(p-1)!\begin{pmatrix}n\\ p-1\end{pmatrix}|X|^2$$. Concerning the second sum, we make use of the identity $\sum _{i=1}^{n+m} |(\partial x_i)^T\wedge \omega |^2=(n-p)|\omega |^2$, and deduce that the second sum is equal to

$$\begin{aligned} (n-p+2)\sum _{i_2,\ldots ,i_p} |X\lrcorner \left( (\partial x_{i_2})^T\wedge \ldots \wedge (\partial x_{i_p})^T\right) |^2. \end{aligned}$$

After using $X=\sum _{i=1}^{n+m} g(X,(\partial x_i)^T) (\partial x_i)^T$, the last sum reduces to

$$\begin{aligned} -2\sum _{i_2,\ldots ,i_p} |X\lrcorner \left( (\partial x_{i_2})^T\wedge \ldots \wedge (\partial x_{i_p})^T\right) |^2. \end{aligned}$$

Here, we conclude that

$$\begin{aligned}&\sum _{i_1,\ldots ,i_p} |X\lrcorner \left( (\partial x_{i_1})^T\wedge \ldots \wedge (\partial x_{i_p})^T\right) |^2=(p-1)!\begin{pmatrix}n\\ p-1\end{pmatrix}|X|^2 \\&\quad +(n-p)\sum _{i_2,\ldots ,i_p} |X\lrcorner \left( (\partial x_{i_2})^T\wedge \ldots \wedge (\partial x_{i_p})^T\right) |^2. \end{aligned}$$

Thus, by induction, we deduce that

3.10 $$\begin{aligned}&\sum _{i_1,\ldots ,i_p}|X\lrcorner \left( (\partial x_{i_1})^T\wedge \ldots \wedge (\partial x_{i_p})^T\right) |^2 =|X|^2\left( (p-1)!\begin{pmatrix}n\\ p-1\end{pmatrix}\right. \nonumber \\&\quad \left. +(n-p)(p-2)!\begin{pmatrix}n\\ p-2\end{pmatrix}\right. \nonumber \\&\quad \left. +(n-p)(n-p+1)(p-3)!\begin{pmatrix}n\\ p-3\end{pmatrix}+\ldots \right) . \end{aligned}$$

After some algebraic manipulations, Equality (3.10) reduces to the following

$$\begin{aligned} |X|^2&\frac{n!}{(n-p-1)!}\sum _{k=0}^{p-1}\frac{1}{(n-p+k)(n-p+k+1)} \\&=|X|^2 \frac{n!}{(n-p-1)!}\sum _{k=0}^{p-1}\left( \frac{1}{n-p+k}-\frac{1}{n-p+k+1}\right) \\&=|X|^2 \frac{n!}{(n-p-1)!} \frac{p}{n(n-p)}\\&=p!\begin{pmatrix}n-1\\ p-1\end{pmatrix}|X|^2. \end{aligned}$$

This finishes the proof of the Lemma. $\square$

Using Lemma 3.1 with $X=df$ and Equation (3.8), we deduce that the sum over $i_1,\ldots ,i_p$ of Eq. (3.9) gives the following

$$\begin{aligned} \sum _{i_1,\ldots ,i_p}|\delta _f\left( (\partial x_{i_1})^T\wedge \ldots \wedge (\partial x_{i_p})^T\right) |^2=p!||T^{[p-1]}||^2+p!\begin{pmatrix}n-1\\ p-1\end{pmatrix}|df|^2. \end{aligned}$$

Hence, by plugging Eq. (3.7) into Inequality (3.6) we get the estimate

$$\begin{aligned} \lambda '_{1,p,f}\le \frac{1}{\textrm{Vol}_f(M)}\int _M \left( pn|H|^2-\frac{p(p-1)}{n(n-1)}\textrm{Scal}^M+\frac{p}{n}|df|^2\right) d\mu _f \end{aligned}$$

completing the proof of Theorem 1.1. $\square$

Before we turn to the proof of Theorem 1.3, we recall the following powerful results from [3] in which the authors show the following:

### Theorem 3.2

[3, Thm. 2]. Let $\mathcal {H}$ be a complex Hilbert space with a given inner product $\langle \cdot ,\cdot \rangle$. Let $A:\mathcal {D}\subset \mathcal {H}\rightarrow \mathcal {H}$ be a self-adjoint operator defined on a dense domain $\mathcal {D}$ which is semi-bounded below and has a discrete spectrum $\lambda _1\le \lambda _2\le \lambda _3\ldots$. Let $\{B_k:A(\mathcal {D})\rightarrow \mathcal {H}\}_{k=1}^N$ be a collection of symmetric operators which leave $\mathcal {D}$ invariant and let $\{u_i\}_{i=1}^\infty$ be the normalized eigenvectors of *A*, $u_i$ corresponding to $\lambda _i$. This family is assumed to be an orthonormal basis of $\mathcal {H}$. Let *g* be a nonnegative and nondecreasing function of the eigenvalues $\{\lambda _i\}_{i=1}^m$. Then we have the inequality

$$\begin{aligned}{} & {} \sum _{i=1}^m\sum _{k=1}^N (\lambda _{m+1}-\lambda _i)^2g(\lambda _i)\langle [A,B_k]u_i,B_k u_i\rangle \\{} & {} \quad \le \sum _{i=1}^m\sum _{k=1}^N (\lambda _{m+1}-\lambda _i)g(\lambda _i)||[A,B_k]u_i||^2. \end{aligned}$$

Here $[A,B]:=AB-BA$ is the commutator of the two operators *A* and *B*.

As a corollary of this result and by taking $g(\lambda )=(\lambda _{m+1}-\lambda )^{\alpha -2}$ for $\alpha \le 2$, we get the following inequality [3, Cor. 3]

3.11 $$\begin{aligned} \sum _{i=1}^m\sum _{k=1}^N(\lambda _{m+1}-\lambda _i)^\alpha \langle [A,B_k]u_i,B_k u_i\rangle \le \sum _{i=1}^m\sum _{k=1}^N (\lambda _{m+1}-\lambda _i)^{\alpha -1}||[A,B_k]u_i||^2. \end{aligned}$$

In the same paper, the authors deal with another type of inequalities treated by Harell and Stubbe [17]. For this, we say that a real function *f* satisfies condition (H1) if there exists a function *r*(*x*) such that

$$\begin{aligned} \frac{f(x)-f(y)}{x-y}\ge \frac{r(x)+r(y)}{2}\qquad \text {(H1)}. \end{aligned}$$

An example of such a function *f* is whenever $f'$ is concave, in this case $r=f'$. In [3], Ashbaugh and Hermi prove the following:

### Theorem 3.3

[3, Thm. 7]. Under the same assumptions as in the previous theorem, and if *f* is a function satisfying the condition (H1), we have

$$\begin{aligned} \sum _{i=1}^m f(\lambda _i)\left( \sum _{k=1}^N \langle [A,B_k]u_i,B_k u_i\rangle \right) \le -\frac{1}{2}\sum _{i=1}^m r(\lambda _i)\left( \sum _{k=1}^N ||[A,B_k]u_i||^2\right) +\mathfrak {R}, \end{aligned}$$

where

$$\begin{aligned} \mathfrak {R}=\sum _{k=1}^N\sum _{i=1}^m\sum _{j=m+1}^\infty |\langle [A,B_k]u_i, u_j\rangle |^2\left( \frac{f(\lambda _i)}{\lambda _{m+1}-\lambda _i}+\frac{1}{2}r(\lambda _i)\right) . \end{aligned}$$

For the particular case, when $f(\lambda )=(\lambda _{m+1}-\lambda )^\alpha$ with $\alpha \ge 2$, they deduce the following inequality [3, Cor. 8]

3.12 $$\begin{aligned} \sum _{i=1}^m\sum _{k=1}^N(\lambda _{m+1}-\lambda _i)^\alpha \langle [A,B_k]u_i,B_k u_i\rangle \le \frac{\alpha }{2}\sum _{i=1}^m\sum _{k=1}^N (\lambda _{m+1}-\lambda _i)^{\alpha -1}||[A,B_k]u_i||^2. \end{aligned}$$

### Proof of Theorem 1.3

In the following we will use Inequalities (3.11) and (3.12) in the case of the drifting Hodge Laplacian defined on a manifold with boundary. Recall that on a compact Riemannian manifold $(\Omega ,g)$ with boundary $\partial \Omega$ the Dirichlet problem on differential *p*-forms is given by

3.13 $$\begin{aligned} \left\{ \begin{array}{ll} \Delta _f\omega =\lambda _{p,f}\omega \,\, &{}\quad \text {on}\,\, \Omega \\ \omega =0 \,\, &{}\quad \text {on}\,\, \partial \Omega . \end{array}\right. \end{aligned}$$

We now take $A=\Delta _f$ and $B_k$ a function on $\Omega$, which we will denote by *G*, in Inequalities (3.11) and (3.12). We follow closely the computations done in [20]. Throughout the proof we choose an orthonormal frame $\{e_i\}_{i=1,\ldots ,n}$ of *TM* such that $\nabla e_i=0$ at a fixed point. First, using Eq. (2.6), we compute

$$\begin{aligned}{}[\Delta _f,G]\omega= & {} [\nabla _f^*\nabla ,G]\omega \\= & {} \left( -\sum _{i=1}^n\nabla _{e_i}\nabla _{e_i}+\nabla _{df}\right) (G\omega )-G\nabla _f^*\nabla \omega \\= & {} (\Delta _f G)\omega -2\nabla _{dG}\omega . \end{aligned}$$

Here, we recall that $\Delta _f G=\Delta G+g(df,dG)$. Therefore, we get that

3.14 $$\begin{aligned} \int _\Omega \langle [\Delta _f,G]\omega ,G\omega \rangle d\mu _f= & {} \int _\Omega G(\Delta _f G)|\omega |^2 d\mu _f -2\int _\Omega G\langle \nabla _{dG}\omega ,\omega \rangle d\mu _f\nonumber \\= & {} \int _\Omega G(\Delta _f G)|\omega |^2 d\mu _f-\frac{1}{2}\int _\Omega \langle dG^2,d(|\omega |^2)\rangle d\mu _f\nonumber \\= & {} \int _\Omega G(\Delta _f G)|\omega |^2 d\mu _f-\frac{1}{2}\int _\Omega |\omega |^2(\Delta _f G^2) d\mu _f\nonumber \\= & {} \int _\Omega |\omega |^2|dG|^2 d\mu _f. \end{aligned}$$

In the last equality, we use the fact that $\Delta _f G^2=2\,G\Delta _f G-2|dG|^2$ which can be shown by a straightforward computation. Also, we have that

3.15 $$\begin{aligned} \int _\Omega |[\Delta _f,G]\omega |^2 d\mu _f{} & {} =\int _\Omega (\Delta _f G)^2|\omega |^2 d\mu _f+4\int _\Omega |\nabla _{dG}\omega |^2 d\mu _f\nonumber \\{} & {} \quad -4\int _\Omega \Delta _f G\langle \omega , \nabla _{dG}\omega \rangle d\mu _f.\nonumber \\ \end{aligned}$$

In the following, we will assume that the manifold $\Omega$ is a domain in a complete Riemannian manifold $(M^n,g)$ that is isometrically immersed into the Euclidean space $\mathbb {R}^{n+m}$ endowed with its canonical metric. We choose in the above formulas the function *G* to be $G=x_l, l=1,\ldots ,n+m$ the components of the immersion $X=(x_1,\ldots ,x_{n+m})$. Recall that $(\partial x_l)^T=d(x_l\circ \iota )$ where $\iota$ is the isometric immersion. In addition, $\omega =\omega _i$ are eigenforms of the problem (3.13) associated to the eigenvalues $\lambda _{i,p,f}$ that are chosen to be of $L^2$-norm equal to 1. The computation done in (3.14) gives after summing over *l* that

$$\begin{aligned} \sum _{l=1}^{m+n}\int _\Omega \langle [\Delta _f,x_l]\omega _i,x_l\omega _i\rangle d\mu _f =\sum _{l=1}^{m+n}\int _\Omega |dx_l|^2|\omega _i|^2 d\mu _f=n. \end{aligned}$$

Recall here that $\sum _{l=1}^{n+m} |dx_l|^2=n$. By taking $G=x_l$ in Eq. (3.15) and summing over *l* yields

$$\begin{aligned} \sum _{l=1}^{n+m}\int _\Omega |[\Delta _f,x_l]\omega _i|^2 d\mu _f= & {} \sum _{l=1}^{n+m}\int _\Omega (\Delta _f x_l)^2|\omega _i|^2 d\mu _f+4\sum _{l=1}^{n+m}\int _\Omega |\nabla _{dx_l}\omega _i|^2 d\mu _f \\{} & {} -4\sum _{l=1}^{n+m}\int _\Omega (\Delta _f x_l)\langle \omega _i, \nabla _{dx_l}\omega _i\rangle d\mu _f.\\= & {} \sum _{l=1}^{n+m}\int _\Omega \left( \Delta x_l+g(df,dx_l)\right) ^2|\omega _i|^2 d\mu _f+4\int _\Omega |\nabla _{dx_l}\omega _i|^2 d\mu _f\\ {}{} & {} -4\int _\Omega \left( \Delta x_l+g(df,dx_l)\right) \langle \omega _i, \nabla _{dx_l}\omega _i\rangle d\mu _f\\= & {} \int _\Omega (n^2|H|^2+|df|^2+2ng(df,H))|\omega _i|^2 d\mu _f+4\int _\Omega |\nabla \omega _i|^2 d\mu _f\\{} & {} -2\int _\Omega (n g(H,d|\omega _i|^2)+g(df,d|\omega _i|^2)) d\mu _f\\= & {} \int _\Omega (n^2|H|^2+|df|^2)|\omega _i|^2 d\mu _f+4\int _\Omega |\nabla \omega _i|^2 d\mu _f \\{} & {} -2\int _\Omega g(df,d|\omega _i|^2) d\mu _f. \end{aligned}$$

Here, we used that the mean curvature of the immersion is given by $H=\frac{1}{n}(\Delta x_1,\ldots , \Delta x_{n+m})$. Applying the Bochner-Weitzenböck formula (2.6) to $\omega _i$ and taking the scalar product with $\omega _i$ itself, the above equality acquires the form:

$$\begin{aligned} {}{} & {} {}\sum _{l=1}^{n+m}\int _\Omega |[\Delta _f,x_l]\omega _i|^2 d\mu _f\\{}{} & {} {} \quad = \int _\Omega (n^2|H|^2+|df|^2)|\omega _i|^2 d\mu _f \\{}{} & {} {} \qquad +4\left( \lambda _{i,p,f}-\frac{1}{2}\int _\Omega \Delta _f(|\omega _i|^2) d\mu _f-\int _\Omega \langle (\mathfrak {B}^{[p]}+T_f^{[p]})\omega _i,\omega _i\rangle d\mu _f\right) \\{}{} & {} {} \qquad -2\int _\Omega (\Delta _f f) |\omega _i|^2 d\mu _f\\{}{} & {} {} \quad =\int _\Omega (n^2|H|^2+|df|^2)|\omega _i|^2 d\mu _f \\{}{} & {} {} \qquad +4\left( \lambda _{i,p,f} -\frac{1}{2}\int _{\partial \Omega }\frac{\partial }{\partial \nu }(|\omega _i|^2) d\mu _f -\int _\Omega \langle (\mathfrak {B}^{[p]}+T_f^{[p]})\omega _i,\omega _i\rangle d\mu _f\right) \\{}{} & {} {} \qquad -2\int _\Omega (\Delta f+|df|^2)|\omega _i|^2 d\mu _f\\{}{} & {} {} \quad =\int _\Omega n^2|H|^2|\omega _i|^2 d\mu _f+4\left( \lambda _{i,p,f}-\int _\Omega \langle (\mathfrak {B}^{[p]}+T_f^{[p]})\omega _i,\omega _i\rangle d\mu _f\right) \\{}{} & {} {} \qquad -\int _\Omega (2\Delta f+|df|^2)|\omega _i|^2 d\mu _f.\\ \end{aligned}$$

Inserting the above equations into (3.11) and (3.12) completes the proof of Theorem 1.3. $\square$

### Proof of Theorem 1.7

First of all, let *u* be the function given by

$$\begin{aligned} u^{i_1,\ldots ,i_{p+1}}=\langle \partial x_{i_1}\wedge \ldots \wedge \partial x_{i_{p+1}},\nu \wedge \omega \rangle \end{aligned}$$

where $\omega$ is a *p*-form on *M* and $\nu$ is the inward unit normal vector field. Throughout the proof we will denote by $\nabla ^{\mathbb {R}^{n+1}}$ the connection on $\mathbb {R}^{n+1}$ and by $\nabla$ the connection on *M*, as well as for ${d^{\mathbb {R}^{n+1}}}$ and *d*. To simplify the notations, we will denote $u^{i_1,\ldots ,i_{p+1}}$ by *u* in the following technical lemma (see [21, Lem. 3.1] for $p=1$).

### Lemma 3.4

Suppose that *M* is a *f*-minimal hypersurface of the weighted manifold $(\mathbb {R}^{n+1},g=\textrm{can}, e^{-f}dv_g)$. Then, we have

$$\begin{aligned} L_fu= & {} -\langle \partial x_{i_1}\wedge \ldots \wedge \partial x_{i_{p+1}},({\nabla _\nu ^{\mathbb {R}^{n+1}}} {d^{\mathbb {R}^{n+1}}}f)^T\wedge \omega \rangle \\{} & {} +2\langle \partial x_{i_1}\wedge \ldots \wedge \partial x_{i_{p+1}},{\textbf{II}}(e_i)\wedge \nabla _{e_i}\omega \rangle \\{} & {} -\langle \partial x_{i_1}\wedge \ldots \wedge \partial x_{i_{p+1}},\nu \wedge ({\textbf{II}}^2)^{[p]}\omega \rangle +\langle \partial x_{i_1}\wedge \ldots \wedge \partial x_{i_{p+1}},\nu \wedge \Delta _f\omega \rangle \\{} & {} +\langle \partial x_{i_1}\wedge \ldots \wedge \partial x_{i_{p+1}},\nu \wedge ({\textbf{II}}^{[p]})^2\omega \rangle -\langle \partial x_{i_1}\wedge \ldots \wedge \partial x_{i_{p+1}},\nu \wedge {{\widetilde{T}}}_f^{[p]}\omega \rangle \\{} & {} -\textrm{Hess}^{\mathbb {R}^{n+1}} f(\nu ,\nu )u. \end{aligned}$$

Here ${{\widetilde{T}}}_f^{[p]}$ is defined in the same way as in Sect. 2.2 on $\mathbb {R}^{n+1}$.

### Proof

For any $X\in TM$, we have

$$\begin{aligned} X(u)=-\langle \partial x_{i_1}\wedge \ldots \wedge \partial x_{i_{p+1}},{\textbf{II}}(X)\wedge \omega \rangle +\langle \partial x_{i_1}\wedge \ldots \wedge \partial x_{i_{p+1}},\nu \wedge \nabla _X\omega \rangle . \end{aligned}$$

Here, we used the fact that $(\nabla ^{\mathbb {R}^{n+1}}_X\omega )^T=\nabla _X\omega$, since $\omega$ is a differential form on *M*. Differentiating again with respect to *X* yields

$$\begin{aligned} X(X(u))= & {} -\langle \partial x_{i_1}\wedge \ldots \wedge \partial x_{i_{p+1}},\nabla ^{\mathbb {R}^{n+1}}_X {\textbf{II}}(X)\wedge \omega \rangle \\{} & {} -\langle \partial x_{i_1}\wedge \ldots \wedge \partial x_{i_{p+1}},{\textbf{II}}(X)\wedge \nabla ^{\mathbb {R}^{n+1}}_X\omega \rangle \\{} & {} -\langle \partial x_{i_1}\wedge \ldots \wedge \partial x_{i_{p+1}}, {\textbf{II}}(X)\wedge \nabla _X\omega \rangle \\{} & {} +\langle \partial x_{i_1}\wedge \ldots \wedge \partial x_{i_{p+1}}, \nu \wedge \nabla ^{\mathbb {R}^{n+1}}_X\nabla _X\omega \rangle \\= & {} -\langle \partial x_{i_1}\wedge \ldots \wedge \partial x_{i_{p+1}},\nabla _X {\textbf{II}}(X)\wedge \omega \rangle \\{} & {} -|{\textbf{II}}(X)|^2\langle \partial x_{i_1}\wedge \ldots \wedge \partial x_{i_{p+1}},\nu \wedge \omega \rangle \\{} & {} -2\langle \partial x_{i_1}\wedge \ldots \wedge \partial x_{i_{p+1}},{\textbf{II}}(X)\wedge \nabla _X\omega \rangle \\{} & {} -\langle \partial x_{i_1}\wedge \ldots \wedge \partial x_{i_{p+1}}, {\textbf{II}}(X)\wedge \nu \wedge {\textbf{II}}(X)\lrcorner \omega \rangle \\{} & {} +\langle \partial x_{i_1}\wedge \ldots \wedge \partial x_{i_{p+1}}, \nu \wedge \nabla _X\nabla _X\omega \rangle . \end{aligned}$$

In the last part, we used the Gauss formula and the identity $\nu \lrcorner \nabla ^{\mathbb {R}^{n+1}}_X\omega ={\textbf{II}}(X)\lrcorner \omega$. Now tracing over an orthonormal frame $\{e_i\}$ of *TM* gives that

$$\begin{aligned} \Delta _f u= & {} \Delta u+g(df,du)\\= & {} -\langle \partial x_{i_1}\wedge \ldots \wedge \partial x_{i_{p+1}}, (\delta {\textbf{II}})\wedge \omega \rangle +|{\textbf{II}}|^2\langle \partial x_{i_1}\wedge \ldots \wedge \partial x_{i_{p+1}},\nu \wedge \omega \rangle \\{} & {} +2\langle \partial x_{i_1}\wedge \ldots \wedge \partial x_{i_{p+1}},{\textbf{II}}(e_i)\wedge \nabla _{e_i}\omega \rangle -\langle \partial x_{i_1}\wedge \ldots \wedge \partial x_{i_{p+1}},\nu \wedge ({\textbf{II}}^2)^{[p]}\omega \rangle \\{} & {} -\langle \partial x_{i_1}\wedge \ldots \wedge \partial x_{i_{p+1}}, \nu \wedge \nabla _{e_i}\nabla _{e_i}\omega \rangle -\langle \partial x_{i_1}\wedge \ldots \wedge \partial x_{i_{p+1}},{\textbf{II}}(df)\wedge \omega \rangle \\ {}{} & {} +\langle \partial x_{i_1}\wedge \ldots \wedge \partial x_{i_{p+1}},\nu \wedge \nabla _{df}\omega \rangle .\\= & {} n\langle \partial x_{i_1}\wedge \ldots \wedge \partial x_{i_{p+1}}, dH\wedge \omega \rangle +|{\textbf{II}}|^2u+2\langle \partial x_{i_1}\wedge \ldots \wedge \partial x_{i_{p+1}},{\textbf{II}}(e_i)\wedge \nabla _{e_i}\omega \rangle \\ {}{} & {} -\langle \partial x_{i_1}\wedge \ldots \wedge \partial x_{i_{p+1}},\nu \wedge ({\textbf{II}}^2)^{[p]}\omega \rangle +\langle \partial x_{i_1}\wedge \ldots \wedge \partial x_{i_{p+1}}, \nu \wedge \nabla _f^*\nabla \omega \rangle \\ {}{} & {} -\langle \partial x_{i_1}\wedge \ldots \wedge \partial x_{i_{p+1}},{\textbf{II}}(df)\wedge \omega \rangle . \end{aligned}$$

In the last equality, we used the fact that $\delta {\textbf{II}}=-ndH$ and the expression of $\nabla _f^*\nabla$. Now, combining Equation (4.5) with the Bochner-Weitzenböck formula (2.6) combined with (4.5) that is shown in the next section, the above equality reduces to

$$\begin{aligned} \Delta _f u= & {} n\langle \partial x_{i_1}\wedge \ldots \wedge \partial x_{i_{p+1}}, dH\wedge \omega \rangle +|{\textbf{II}}|^2u+2\langle \partial x_{i_1}\wedge \ldots \wedge \partial x_{i_{p+1}},{\textbf{II}}(e_i)\wedge \nabla _{e_i}\omega \rangle \\{} & {} -\langle \partial x_{i_1}\wedge \ldots \wedge \partial x_{i_{p+1}},\nu \wedge ({\textbf{II}}^2)^{[p]}\omega \rangle +\langle \partial x_{i_1}\wedge \ldots \wedge \partial x_{i_{p+1}}, \nu \wedge \Delta _f\omega \rangle \\{} & {} +\langle \partial x_{i_1}\wedge \ldots \wedge \partial x_{i_{p+1}}, \nu \wedge \left( ({\textbf{II}}^{[p]})^2-nH {\textbf{II}}^{[p]}-T_f^{[p]}\right) \omega \rangle \\{} & {} -\langle \partial x_{i_1}\wedge \ldots \wedge \partial x_{i_{p+1}},{\textbf{II}}(df)\wedge \omega \rangle . \end{aligned}$$

Now, for a *f*-minimal hypersurface, we write

$$\begin{aligned} d^{\mathbb {R}^{n+1}}f=df+\frac{\partial f}{\partial \nu }\nu =df-nH\nu . \end{aligned}$$

Hence, by differentiating along a vector $X\in TM$, we get that

$$\begin{aligned} (\nabla ^{\mathbb {R}^{n+1}}_X d^{\mathbb {R}^{n+1}}f)^T=\nabla _X df+nH {\textbf{II}}(X). \end{aligned}$$

Therefore, we find

$$\begin{aligned} ({\widetilde{T}}_f^{[p]}\omega )^T=\sum _{i=1}^n e_i\wedge (\nabla ^{\mathbb {R}^{n+1}}_{e_i} d^{\mathbb {R}^{n+1}}f)^T\lrcorner \omega =T_f^{[p]}\omega +nH {\textbf{II}}^{[p]}\omega . \end{aligned}$$

Also, we have that

$$\begin{aligned} ndH=-d\left( \frac{\partial f}{\partial \nu }\right) =-(\nabla ^{\mathbb {R}^{n+1}}_\nu d^{\mathbb {R}^{n+1}}f)^T+{\textbf{II}}(df). \end{aligned}$$

Hence, plugging these computations into the expression of $\Delta _f u$, we arrive at

$$\begin{aligned} \Delta _f u= & {} -\langle \partial x_{i_1}\wedge \ldots \wedge \partial x_{i_{p+1}}, (\nabla ^{\mathbb {R}^{n+1}}_\nu {d^{\mathbb {R}^{n+1}}}f)^T \wedge \omega \rangle \\{} & {} +|{\textbf{II}}|^2u+2\langle \partial x_{i_1}\wedge \ldots \wedge \partial x_{i_{p+1}},{\textbf{II}}(e_i)\wedge \nabla _{e_i}\omega \rangle \\ {}{} & {} -\langle \partial x_{i_1}\wedge \ldots \wedge \partial x_{i_{p+1}},\nu \wedge ({\textbf{II}}^2)^{[p]}\omega \rangle +\langle \partial x_{i_1}\wedge \ldots \wedge \partial x_{i_{p+1}}, \nu \wedge \Delta _f\omega \rangle \\ {}{} & {} +\langle \partial x_{i_1}\wedge \ldots \wedge \partial x_{i_{p+1}}, \nu \wedge ({\textbf{II}}^{[p]})^2\omega \rangle -\langle \partial x_{i_1}\wedge \ldots \wedge \partial x_{i_{p+1}}, \nu \wedge {{\widetilde{T}}}_f^{[p]}\omega \rangle . \end{aligned}$$

The proof of the lemma then follows from the expression of $L_f$. $\square$

In order to complete the proof of Theorem 1.7, we proceed as in [21]. Let $\{\varphi _j\}$ be an $L^2(e^{-f}dv_g)$-orthonormal basis of eigenfunctions of $L_f$ associated to $\lambda _j(L_f)$. Let $E^d$ be the sum of the first *d*-eigenspaces of $\Delta _f$:

$$\begin{aligned} E^d={\mathop {\oplus }\limits _{j=1}^d} V_{\Delta _f}(\lambda _{j,p,f}). \end{aligned}$$

For all $l\ge 2$, we consider the following system of equations

$$\begin{aligned} \int _M u^{i_1,\ldots ,i_{p+1}}\varphi _1 d\mu _f=\ldots =\int _M u^{i_1,\ldots ,i_{p+1}}\varphi _{l-1} d\mu _f=0, \end{aligned}$$

where we recall that $u^{i_1,\ldots ,i_{p+1}}=\langle \partial x_{i_1}\wedge \ldots \partial x_{i_{p+1}}, \nu \wedge \omega \rangle$. This system consists of $$\begin{pmatrix}n+1\\ p+1\end{pmatrix}(l-1)$$ homogeneous linear equations in the variable $\omega \in E^d$. Hence, if we take $$d= \begin{pmatrix}n+1\\ p+1\end{pmatrix}(l-1)+1$$, we can find a non-trivial $\omega \in E^d$ which is orthogonal to the first $(l-1)$-eigenfunctions of $L_f$. Therefore, we deduce that

$$\begin{aligned} \lambda _l(L_f)\int _M (u^{i_1,\ldots ,i_{p+1}})^2 d\mu _f\le \int _M u^{i_1,\ldots ,i_{p+1}} L_f(u^{i_1,\ldots ,i_{p+1}})d\mu _f. \end{aligned}$$

Summing over $i_1<\ldots <i_{p+1}$, we first have for the l.h.s. that

$$\begin{aligned} \sum _{i_1<\ldots <i_{p+1}} (u^{i_1,\ldots ,i_{p+1}})^2=|\nu \wedge \omega |^2=|\omega |^2. \end{aligned}$$

For the r.h.s. of the previous inequality, we use the previous lemma to get that

$$\begin{aligned}{} & {} \sum _{i_1<\ldots <i_{p+1}} u^{i_1,\ldots ,i_{p+1}} L_f(u^{i_1,\ldots ,i_{p+1}})\\{} & {} \quad =2\langle \nu \wedge \omega ,{\textbf{II}}(e_i)\wedge \nabla _{e_i}\omega \rangle -\langle \nu \wedge \omega ,\nu \wedge ({\textbf{II}}^2)^{[p]}\omega \rangle \\{} & {} \qquad +\langle \nu \wedge \omega ,\nu \wedge \Delta _f\omega \rangle +\langle \nu \wedge \omega ,\nu \wedge ({\textbf{II}}^{[p]})^2\omega \rangle \\{} & {} \qquad -\langle \nu \wedge \omega ,\nu \wedge {{\widetilde{T}}}_f^{[p]}\omega \rangle -\textrm{Hess}^{\mathbb {R}^{n+1}} f(\nu ,\nu )|\nu \wedge \omega |^2\\{} & {} \quad =-\langle ({\textbf{II}}^2)^{[p]}\omega ,\omega \rangle +\langle \Delta _f\omega ,\omega \rangle +\langle ({\textbf{II}}^{[p]})^2\omega ,\omega \rangle \\{} & {} \qquad -\langle {{\widetilde{T}}}_f^{[p]}\omega ,\omega \rangle - \textrm{Hess}^{\mathbb {R}^{n+1}} f(\nu ,\nu )|\omega |^2. \end{aligned}$$

Hence, we deduce that

$$\begin{aligned}{} & {} \lambda _l(L_f)\int _M |\omega |^2 d\mu _f\\{} & {} \quad \le \int _M \big (-\langle ({\textbf{II}}^2)^{[p]}\omega ,\omega \rangle +\langle \Delta _f\omega ,\omega \rangle +\langle ({\textbf{II}}^{[p]})^2\omega ,\omega \rangle -\langle {{\widetilde{T}}}_f^{[p]}\omega ,\omega \rangle \\{} & {} \qquad -\textrm{Hess}^{\mathbb {R}^{n+1}} f(\nu ,\nu )|\omega |^2\big )d\mu _f\\{} & {} \quad \le \int _M \left( -\langle ({\textbf{II}}^2)^{[p]}\omega ,\omega \rangle +\langle \Delta _f\omega ,\omega \rangle +\langle ({\textbf{II}}^{[p]})^2\omega ,\omega \rangle -(p+1)a|\omega |^2\right) d\mu _f. \end{aligned}$$

In the last equality, we used the fact that any eigenvalue of the operator ${{\widetilde{T}}}_f^{[p]}$ is the sum of *p*-distinct eigenvalues of $\textrm{Hess}^{\mathbb {R}^{n+1}}f$. Now, we have that

$$\begin{aligned} \int _M\langle \Delta _f\omega ,\omega \rangle d\mu _f\le \lambda _{d(l),p,f}\int _M|\omega |^2d\mu _f, \end{aligned}$$

since $\omega \in E^{d(l)}$. Also, we have that

$$\begin{aligned} -\langle ({\textbf{II}}^2)^{[p]}\omega ,\omega \rangle +\langle ({\textbf{II}}^{[p]})^2\omega ,\omega \rangle= & {} -\sum _{i=1}^n\langle {\textbf{II}}(e_i)\wedge \textbf{II}(e_i)\lrcorner \omega ,\omega \rangle +\left| \sum _{i=1}^n e_i\wedge \textbf{II}(e_i)\lrcorner \omega \right| ^2\\= & {} -\sum _{i,j=1}^n\langle e_j\lrcorner \textbf{II}(e_i)\lrcorner \omega ,e_i\lrcorner \textbf{II}(e_j)\lrcorner \omega \rangle \\\le & {} \gamma _M p(p-1)|\omega |^2. \end{aligned}$$

Finally, we deduce that

$$\begin{aligned} \lambda _l(L_f) \le \lambda _{d(l),p,f}-(p+1)a+\gamma _M p(p-1), \end{aligned}$$

which finishes the proof of the theorem. $\square$

### Proof of Corollaries 1.8 and 1.9

Let $l_0$ be the largest integer such that $d(l_0)\le \beta$. Thus,

$$\begin{aligned} \textrm{Ind}_f(M)\ge l_0. \end{aligned}$$

Now as we have that $$d\left( \frac{1}{\begin{pmatrix}n+1\\ p+1\end{pmatrix}}\beta +1-\frac{1}{\begin{pmatrix}n+1\\ p+1\end{pmatrix}}\right) \le \beta $$, then

$$\begin{aligned} l_0\ge \left[ \frac{1}{\begin{pmatrix}n+1\\ p+1\end{pmatrix}}\beta +1-\frac{1}{\begin{pmatrix}n+1\\ p+1\end{pmatrix}}\right] \ge \frac{1}{\begin{pmatrix}n+1\\ p+1\end{pmatrix}}\beta . \end{aligned}$$

This proves the first corollary. To prove the second one, we take as before

$$\begin{aligned} l_0=\left[ \frac{1}{\begin{pmatrix}n+1\\ p+1\end{pmatrix}}b_p(M)+1-\frac{1}{\begin{pmatrix}n+1\\ p+1\end{pmatrix}}\right] , \end{aligned}$$

then, we clearly have that $d(l_0)\le b_p(M)$. Therefore, $\lambda _{d(l_0),p,f}=0$. In the case of a self-shrinker, we recall that for $f=\frac{|X|^2}{2}$ the hypersurface *M* is *f*-minimal and that $\textrm{Hess}^{\mathbb {R}^{n+1}} f=g=\textrm{can}$. Hence with the assumption $p(p-1)\gamma _M\le p+1$, we deduce that $\lambda _{l_0}(L_f)\le 0$. Finally, we get that the index is at least $n+1+l_0$ by the fact that $L_f$ has at least $n+1$ eigenvalues equal to $-1$. This finishes the proof. $\square$

## Geometric Applications of the Main Results

In this section we will provide several geometric applications of Theorem 1.3 by making explicit choices of the function *f*.

First, we will consider the case when *f* is the Riemannian distance function in order to compute explicitly the different terms in the statement of Theorem 1.3. To this end, let $M^n\rightarrow \mathbb {R}^{n+m}$ be an isometric immersion and $\Omega$ a domain in *M*. Consider the function $f:\Omega \rightarrow \mathbb {R}$ given by $f(X)=a\frac{|X|^2}{2}$, where *a* is a real positive number. This particular choice of *f* has important applications in mean curvature flow as described in the introduction of the article. Here, $|\cdot |$ denotes the Euclidean norm in $\mathbb {R}^{n+m}$ and $X=(x_1,\ldots ,x_{n+m})$ are the components of the immersion. In other words, the function *f* is the square of the distance function from the origin point $0\in \mathbb {R}^{n+m}$ to a point $X\in \Omega$. Using the decomposition $X=X^T+X^\perp$, we have that $d^{\mathbb {R}^{n+m}}f=a X$ and therefore, $df=a X^T$. Hence, we deduce that

4.1 $$\begin{aligned} |df|^2=a^2(|X|^2-|X^\perp |^2). \end{aligned}$$

For any $Y\in TM$, we compute

4.2 $$\begin{aligned} \nabla _Ydf= & {} a \nabla _YX^T \nonumber \\= & {} a (\nabla ^{\mathbb {R}^{n+m}}_YX^T)^T\nonumber \\= & {} a(Y-\nabla ^{\mathbb {R}^{n+m}}_YX^\perp )^T\nonumber \\= & {} a\left( Y-\sum _{i=1}^n(\nabla ^{\mathbb {R}^{n+m}}_YX^\perp ,e_i)e_i\right) \nonumber \\= & {} a\left( Y+\sum _{i=1}^n(X^\perp ,\nabla ^{\mathbb {R}^{n+m}}_Ye_i)e_i\right) \nonumber \\= & {} a\left( Y+\sum _{i=1}^n(X^\perp ,\textbf{II}(Y,e_i))e_i\right) \nonumber \\= & {} a(Y+\textbf{II}_{X^\perp }(Y)), \end{aligned}$$

where $\textbf{II}_{X^\perp }$ is defined as in the proof of Theorem 1.1. By tracing (4.2), we deduce that

4.3 $$\begin{aligned} \Delta f=a(-n-n(X,H)). \end{aligned}$$

Also, plugging (4.2) into the expression of $T_f^{[p]}$ yields

$$\begin{aligned} T_f^{[p]}\omega =a(p\omega +\textbf{II}^{[p]}_{X^\perp }\omega ), \end{aligned}$$

where $\textbf{II}^{[p]}_{Z}$ is the canonical extension of $\textbf{II}_{Z}$ to *p*-forms as defined in (3.3). Hence, we deduce

4.4 $$\begin{aligned} \langle T_f^{[p]}\omega ,\omega \rangle =a\left( p|\omega |^2+\langle \textbf{II}^{[p]}_{X^\perp }\omega ,\omega \rangle \right) . \end{aligned}$$

In the following, we will bound the term $\langle \mathfrak {B}^{[p]}\omega _i,\omega _i\rangle$ in Theorem 1.3 by using the results of [38]. For this, recall that A. Savo shows in [38, Thm. 1] that for any isometric immersion $(M^n,g)\rightarrow (N^{n+m},g)$, the Bochner operator $\mathfrak {B}^{[p]}$ on *p*-forms of *M* splits as

$$\begin{aligned} \mathfrak {B}^{[p]}=\mathfrak {B}^{[p]}_{\textrm{ext}}+\mathfrak {B}^{[p]}_{\textrm{res}}, \end{aligned}$$

where $\mathfrak {B}^{[p]}_{\textrm{ext}}$ is the operator defined by

$$\begin{aligned} \mathfrak {B}^{[p]}_{\textrm{ext}}=\sum _{j=1}^{m}\left( \textrm{trace}(\textbf{II}_{\nu _j})\textbf{II}_{\nu _j}^{[p]}-\textbf{II}_{\nu _j}^{[p]}\circ \textbf{II}_{\nu _j}^{[p]}\right) . \end{aligned}$$

Here, $\{\nu _1,\ldots ,\nu _{m}\}$ is a local orthonormal frame of $TM^\perp$, and the operator $\mathfrak {B}^{[p]}_{\textrm{res}}$ is the operator that satisfies

$$\begin{aligned} p(n-p)\gamma _N\le \mathfrak {B}^{[p]}_{\textrm{res}}\le p(n-p)\Gamma _N, \end{aligned}$$

where $\gamma _N$ and $\Gamma _N$ are respectively a lower bound and an upper bound for the curvature operator of *N*. Hence for an isometric immersion $M\rightarrow (\mathbb {R}^{n+m},\textrm{can})$, we deduce that

4.5 $$\begin{aligned} \mathfrak {B}^{[p]}=\mathfrak {B}^{[p]}_{\textrm{ext}}=\textbf{II}_{nH}^{[p]}-\sum _{j=1}^{m}{} \textbf{II}_{\nu _j}^{[p]}\circ \textbf{II}_{\nu _j}^{[p]}. \end{aligned}$$

Inserting Equations (4.1), (4.3), (4.4) and (4.5) in Theorem 1.3, we deduce

### Corollary 4.1

Let $X:(M^n,g)\rightarrow (\mathbb {R}^{n+m},\textrm{can})$ be an isometric immersion and let $f=a\frac{|X|^2}{2}$ where *a* is a real positive number. For any $p\in \{0,\ldots ,n\}$, the eigenvalues of the drifting Hodge Laplacian $\Delta _f$ acting on *p*-forms on a domain $\Omega$ of *M* with Dirichlet boundary conditions satisfy for any $k\ge 1$

$$\begin{aligned} \sum _{i=1}^k(\lambda _{k+1,p,f}-\lambda _{i,p,f})^\alpha\le & {} \frac{4}{n}\sum _{i=1}^k(\lambda _{k+1,p,f}-\lambda _{i,p,f})^{\alpha -1}\big (\lambda _{i,p,f} \\{} & {} -\int _\Omega \langle \textbf{II}_{(aX^\perp +nH)}^{[p]}\omega _i,\omega _i\rangle d\mu _f \\{} & {} +\sum _{j=1}^{m}\int _\Omega |\textbf{II}_{\nu _j}^{[p]}\omega _i|^2d\mu _f-ap+\frac{na}{2} \\{} & {} -\frac{a^2}{4}\int _\Omega |X|^2|\omega _i|^2d\mu _f+\frac{1}{4}\int _\Omega |aX^\perp +nH|^2|\omega _i|^2d\mu _f\big ), \end{aligned}$$

for $\alpha \le 2$. Also, we have

$$\begin{aligned} \sum _{i=1}^k(\lambda _{k+1,p,f}-\lambda _{i,p,f})^\alpha\le & {} \frac{2\alpha }{n}\sum _{i=1}^k(\lambda _{k+1,p,f}-\lambda _{i,p,f})^{\alpha -1}\big (\lambda _{i,p,f} \\{} & {} -\int _\Omega \Bigg \langle \textbf{II}_{(aX^\perp +nH)}^{[p]}\omega _i,\omega _i\Bigg \rangle d\mu _f \\{} & {} +\sum _{j=1}^{m}\int _\Omega |\textbf{II}_{\nu _j}^{[p]}\omega _i|^2d\mu _f-ap+\frac{na}{2}\\{} & {} -\frac{a^2}{4}\int _\Omega |X|^2|\omega _i|^2d\mu _f+\frac{1}{4}\int _\Omega |aX^\perp +nH|^2|\omega _i|^2d\mu _f\Bigg ), \end{aligned}$$

for $$\alpha \ge 2$$.

The result in Corollary 4.1 generalizes the one in [42, Thm. 1.1] when taking $a=\frac{1}{2}, \alpha =2$ and $\Omega =M$ being a domain in $\mathbb {R}^m$. In this case, $\textbf{II}=0$ and we get that

$$\begin{aligned} \sum _{i=1}^k(\lambda _{k+1,p,f}-\lambda _{i,p,f})^2{} & {} \le \frac{4}{n}\sum _{i=1}^k(\lambda _{k+1,p,f}-\lambda _{i,p,f})\\{} & {} \quad \left( \lambda _{i,p,f}-\frac{p}{2}+\frac{n}{4}-\frac{1}{16}{\mathop {\textrm{min}}\limits _\Omega } (|X|^2)\right) . \end{aligned}$$

Moreover, Corollary 4.1 generalizes the result in [42, Thm 1.2] when taking for some integer $l>1$, the number $a=l-1, \alpha =2$ and $(M^n,g)=(\mathbb {R}^{n-l}\times \mathbb {S}^l(1),\langle ,\rangle _{\mathbb {R}^{n-l}}\oplus \langle ,\rangle _{\mathbb {S}^l})$ where $\langle ,\rangle _{\mathbb {S}^l}$ is the standard metric on the unit round sphere $\mathbb {S}^l(1)$ of curvature 1. Indeed, we take the immersion $M\hookrightarrow \mathbb {R}^{n-l}\times \mathbb {R}^{l+1}$ where the second fundamental form is given by the matrix $$\begin{pmatrix} 0&{}\quad 0\\ 0&{}\quad \textrm{Id} \end{pmatrix}$$. Thus, we have $X^\perp =-\nu$ and $nH=\textrm{trace}(\textbf{II})=l\nu$ and, therefore, $aX^\perp +nH=\nu$. Also, using that $\textbf{II}^{[p]}\omega =\sum _{i=1}^n e_i\wedge \textbf{II}(e_i)\lrcorner \omega$ for any *p*-form $\omega$, we deduce by a straightforward computation that

$$\begin{aligned} -\langle \textbf{II}^{[p]}\omega ,\omega \rangle +|\textbf{II}^{[p]}\omega |^2=\sum _{i,j=n-l+1}^n |e_i\lrcorner e_j\lrcorner \omega |^2\le p(p-1)|\omega |^2. \end{aligned}$$

Hence, we get that

$$\begin{aligned}&\sum _{i=1}^k(\lambda _{k+1,p,f}-\lambda _{i,p,f})^2\\&\quad \le \frac{4}{n}\sum _{i=1}^k(\lambda _{k+1,p,f}-\lambda _{i,p,f})\left( \lambda _{i,p,f}+\frac{2n(l-1)+1-4p(l-1)+4p(p-1)}{4}\right. \\&\qquad \left. -\frac{(l-1)^2}{4}{\mathop {\textrm{min}}\limits _\Omega } (|X|^2)\right) . \end{aligned}$$

Corollary 4.1 also generalizes the one in [10] on compact self-shrinkers. In this case, for $a=1$, we get that

$$\begin{aligned}{} & {} \sum _{i=1}^k(\lambda _{k+1,p,f}-\lambda _{i,p,f})^2\\{} & {} \quad \le \frac{4}{n}\sum _{i=1}^k(\lambda _{k+1,p,f}-\lambda _{i,p,f})\Bigg (\lambda _{i,p,f}+\sum _{j=1}^{m}\int _\Omega |\textbf{II}_{\nu _j}^{[p]}\omega _i|^2d\mu _f\\{} & {} \qquad -p+\frac{n}{2}-\frac{1}{4}{\mathop {\textrm{min}}\limits _\Omega } (|X|^2)\Bigg ). \end{aligned}$$

In the last part of this section, we will consider the case when *f* is the distance function from some fixed point in *M* with respect to the Riemannian metric *g* on *M*. For this, fix a point $x_0\in M$ and consider the distance function

$$\begin{aligned} d_{x_0}:\Omega \rightarrow [0,\infty [, d_{x_0}(x)=d(x_0,x) \end{aligned}$$

and $\rho _{x_0}(x):=\frac{1}{2}d_{x_0}(x)^2$. We recall from [31] that the distance function $d_{x_0}$ is smooth in the complement of the cut locus of $x_0$ and that its gradient is of norm 1 almost everywhere. Thus we will choose a domain $\Omega \subset M$ such that it is contained in this complement. Let us recall the comparison theorem [31] (see also [18]). For this, we consider for any $l\in \mathbb {R}$, the function

$$\begin{aligned} H_l(r)=\left\{ \begin{array}{ll} (n-1)\sqrt{l}\,\textrm{cot}(\sqrt{l}r),\,\,\,\, l>0\\ \frac{n-1}{r}, \,\,\,\,l=0\\ (n-1)\sqrt{|l|}\,\textrm{coth}(\sqrt{|l|}r),\,\,\,\, l<0. \end{array}\right. \end{aligned}$$

### Theorem 4.2

[31]. Let $(M^n,g)$ be a complete Riemannian manifold.

1. If $\textrm{Ric}^M\ge (n-1)l$ for some $l\in \mathbb {R}$, then for every $x_0\in M$, the inequalities
  $$\begin{aligned} \Delta d_{x_0}(x)\ge -H_l(d_{x_0}(x)), \quad \text {and}\quad \Delta \rho _{x_0}(x)\ge -(1+d_{x_0}(x)H_l(d_{x_0}(x)), \end{aligned}$$
  hold at smooth points of $d_{x_0}$. Moreover, these inequalities hold on the whole manifold in the sense of distributions.
2. If the sectional curvature satisfies $l_1\le K^M\le l_2$ and $\gamma$ is a minimizing geodesic starting from $x_0\in M$ such that its image is disjoint to the cut locus of $x_0$, then
  $$\begin{aligned} \nabla ^2 d_{x_0}(X,X)\le \frac{H_{l_1}(t)}{n-1}g(X,X), \quad \text {and}\quad \nabla ^2 d_{x_0}(X,X)\ge \frac{H_{l_2}(t)}{n-1}g(X,X), \end{aligned}$$
  for $X\perp \dot{\gamma }(t)$ and $\nabla ^2d_{x_0}(\dot{\gamma }(t),\dot{\gamma }(t))=0,$ for $t\in [0,L]$ for some *L*. As a consequence, one has that
  $$\begin{aligned} \nabla ^2 \rho _{x_0}(X,X)\le \frac{tH_{l_1}(t)}{n-1}g(X,X), \quad \text {and}\quad \nabla ^2 \rho _{x_0}(X,X)\ge \frac{tH_{l_2}(t)}{n-1}g(X,X), \end{aligned}$$
  for $$X\perp \dot{\gamma }(t)$$ and $$\nabla ^2\rho _{x_0}(\dot{\gamma }(t),\dot{\gamma }(t))=1$$.

We will now use Theorem 4.2 to compute the different terms in Theorem 1.3. We begin with the case when $\textrm{Ric}^M\ge (n-1)l$ for some $l\in \mathbb {R}$ on the manifold *M*. By taking $f=a \rho _{x_0}$ where $a>0$, we deduce that the inequalities

$$\begin{aligned} |df|^2=a^2 d_{x_0}^2,\quad \text {and}\quad \Delta f(x)\ge -a\left( 1+d_{x_0}(x)H_l(d_{x_0}(x)\right) \end{aligned}$$

hold in the sense of distributions. Therefore, we deduce from Theorem 1.3 the following

### Corollary 4.3

Let $X:(M^n,g)\rightarrow (\mathbb {R}^{n+m},\textrm{can})$ be an isometric immersion. Assume that $\textrm{Ric}^M\ge (n-1)l$ for some $l\in \mathbb {R}$. Let $x_0$ be fixed point in *M* and $\Omega$ a domain in the complement of the cut locus of $x_0$. Let $f=a\rho _{x_0}$ for some positive real number $a>0$. The eigenvalues of the drifting Hodge Laplacian $\Delta _f$ acting on *p*-forms on $\Omega$ with Dirichlet boundary conditions satisfy for any $k\ge 1$

$$\begin{aligned} \sum _{i=1}^k(\lambda _{k+1,p,f}-\lambda _{i,p,f})^\alpha\le & {} \frac{4}{n}\sum _{i=1}^k(\lambda _{k+1,p,f}-\lambda _{i,p,f})^{\alpha -1}\bigg (\lambda _{i,p,f}-\delta _1+\frac{1}{4}\delta _2\bigg ), \end{aligned}$$

for $\alpha \le 2$, where $\delta _1={\mathop {\textrm{inf}}\limits _\Omega } (\mathfrak {B}^{[p]}+T_f^{[p]})$ and $\delta _2$ is given by

$$\begin{aligned} \delta _2={\mathop {\textrm{sup}}\limits _{x\in \Omega }}\left( n^2|H|^2+2a(1+d_{x_0}(x)H_l(d_{x_0}(x))-a^2d_{x_0}(x)^2\right) . \end{aligned}$$

Also, we have

$$\begin{aligned} \sum _{i=1}^k(\lambda _{k+1,p,f}-\lambda _{i,p,f})^\alpha\le & {} \frac{2\alpha }{n}\sum _{i=1}^k(\lambda _{k+1,p,f}-\lambda _{i,p,f})^{\alpha -1}\bigg (\lambda _{i,p,f}-\delta _1+\frac{1}{4}\delta _2\bigg ), \end{aligned}$$

for $$\alpha \ge 2$$.

In the following, we will consider the case when the sectional curvature of *M* is bounded from below by $l_1$ and from above by $l_2$. We let $\delta _2$ be the number given by

$$\begin{aligned}{} & {} \delta _2\\{} & {} :=\left\{ \begin{array}{ll} {\mathop {\textrm{sup}}\limits _{x\in \Omega }}\left( n^2|H|^2-4a\left( (p-1)\frac{d_{x_0}(x)H_{l_2}(d_{x_0}(x))}{n-1}+1\right) +2a(1+d_{x_0}H_{l_1}(d_{x_0}))-a^2 d_{x_0}^2\right) ,\\ \,\,\,\, l_2\le 0\\ {\mathop {\textrm{sup}}\limits _{x\in \Omega }}\left( n^2|H|^2-4ap\frac{d_{x_0}(x)H_{l_2}(d_{x_0}(x))}{n-1}+2a(1+d_{x_0}H_{l_1}(d_{x_0}))-a^2 d_{x_0}^2\right) , \,\,\,\,l_2>0.\\ \end{array}\right. \end{aligned}$$

We have

### Corollary 4.4

Let $X:(M^n,g)\rightarrow (\mathbb {R}^{n+m},\textrm{can})$ be an isometric immersion. Assume that $l_1\le K^M\le l_2$ for some $l_1,l_2\in \mathbb {R}$. Let $\Omega$ a domain in *M* such that $\Omega$ is contained in the complement of the cut locus of $x_0\in \Omega$. Let $f=a\rho _{x_0}$ for some positive real number $a>0$. For any $p\in \{0,\ldots ,n\}$, the eigenvalues of the drifting Hodge Laplacian $\Delta _f$ acting on *p*-forms on a domain $\Omega$ of *M* with Dirichlet boundary conditions satisfy for any $k\ge 1$

$$\begin{aligned} \sum _{i=1}^k(\lambda _{k+1,p,f}-\lambda _{i,p,f})^\alpha\le & {} \frac{4}{n}\sum _{i=1}^k(\lambda _{k+1,p,f}-\lambda _{i,p,f})^{\alpha -1}\big )\lambda _{i,p,f}-\int _\Omega \langle \textbf{II}_{nH}^{[p]}\omega _i,\omega _i\rangle d\mu _f \\{} & {} +\sum _{j=1}^{m-n}\int _\Omega |\textbf{II}_{\nu _j}^{[p]}\omega _i|^2d\mu _f+\frac{1}{4}\delta _2 \end{aligned}$$

for $\alpha \le 2$. Also, we have

$$\begin{aligned} \sum _{i=1}^k(\lambda _{k+1,p,f}-\lambda _{i,p,f})^\alpha\le & {} \frac{2\alpha }{n}\sum _{i=1}^k(\lambda _{k+1,p,f}-\lambda _{i,p,f})^{\alpha -1}\big )\lambda _{i,p,f}-\int _\Omega \langle \textbf{II}_{nH}^{[p]}\omega _i,\omega _i\rangle d\mu _f\\{} & {} +\sum _{j=1}^{m-n}\int _\Omega |\textbf{II}_{\nu _j}^{[p]}\omega _i|^2d\mu _f+\frac{1}{4}\delta _2 \end{aligned}$$

for $$\alpha \ge 2$$.

### Proof

We proceed as in [36]. At any point $x\in \Omega {\setminus }\{x_0\}$, there is an orthonormal frame $\{e_1(x),\ldots , e_{n-1}(x),e_n=\nabla d_{x_0}(x)\}$ such that $\nabla ^2\rho _{x_0}$ has the eigenvalues $\eta _1(x),\ldots ,\eta _{n-1}(x),1$. By the comparison theorem 4.2, we get that for any $j=1,\ldots ,n-1$

$$\begin{aligned} \frac{d_{x_0}(x)H_{l_2}(d_{x_0}(x))}{n-1}\le \eta _j(x)\le \frac{d_{x_0}(x)H_{l_1}(d_{x_0}(x))}{n-1}. \end{aligned}$$

Therefore, we find that

$$\begin{aligned} |df|^2=a^2 d_{x_0}^2 \quad \text {and}\quad \Delta f\ge -a(1+d_{x_0}H_{l_1}(d_{x_0})). \end{aligned}$$

Recall that the endomorphism $T^{[p]}_f$ is by definition the sum of *p* distinct eigenvalues of $\nabla ^2 f$. Hence, for any *p*-form $\omega$, we get that

$$\begin{aligned} \langle T^{[p]}_f\omega ,\omega \rangle \ge \left\{ \begin{array}{ll} a\left( (p-1)\frac{d_{x_0}(x)H_{l_2}(d_{x_0}(x))}{n-1}+1\right) |\omega |^2,\,\,\,\, l_2\le 0\\ ap\frac{d_{x_0}(x)H_{l_2}(d_{x_0}(x))}{n-1}|\omega |^2, \,\,\,\,l_2>0.\\ \end{array}\right. \end{aligned}$$

This is because of $\frac{d_{x_0}(x)H_{l_2}(d_{x_0}(x))}{n-1}\ge 1$ if $l_2\le 0$ and $\frac{d_{x_0}(x)H_{l_2}(d_{x_0}(x))}{n-1}\le 1$ if $l_2>0$. Replacing all these inequalities in Theorem 1.3, we get the result after combining with Eq. (4.5) for the curvature term.$\square$

## Data Availability

Data sharing not applicable to this article as no datasets were generated or analysed during the current study.
